# A pan‐cancer analysis of prognostic significance and immunological role of lysosomal‐associated membrane protein 3

**DOI:** 10.1111/jcmm.18088

**Published:** 2023-12-26

**Authors:** Xuefei Feng, Lvye Gao, Xinyuan Shen, Mingtai Li, Xiaohui Wang, Yanlong Hao, Jinyan Chen, Yuanfang Zhai, Binbin Zou, Shangman Yao, Yanlin Guo, Ling Zhang

**Affiliations:** ^1^ Department of Pathology, Basic Medical Sciences Center Key Laboratory of Cellular Physiology of Shanxi Medical University Taiyuan China; ^2^ School of Humanities and Social Sciences Shanxi Medical University Taiyuan China

**Keywords:** genetic alteration, immune infiltrates, immunogenetics, LAMP3, pan‐cancer, prognostic significance

## Abstract

Lysosomal dysfunction can drive carcinogenesis. Lysosomal‐associated membrane protein 3 (LAMP3), is a member of the Lysosome Associated Membrane Proteins and is involved in the malignant phenotype such as tumour metastasis and drug resistance, while the mechanisms that regulate the malignant progression of tumour remain vague. Our study aims to provide a more systematic and comprehensive understanding of the role of LAMP3 in the progression of various cancers by various databases.We explored the role of LAMP3 in pan‐cancer using The Cancer Genome Atlas (TCGA) and Genotype‐Tissue Expression (GTEx) database. Multiple online web platforms and software were used for data analysis, including HPA, TIMER, TISIDB, GEPIA, UALCAN, Kaplan–Meier plotter, DAVID and TIGER. The immunohistochemistry was used to quantify the LAMP3 and PD‐L1 expression levels in cancer.High LAMP3 expression was found in most cancers and differentially expressed across molecular and immune subtypes. The expression of LAMP3 was involved in the immune‐associated processes of Antigen processing and presentation, Th17 cell differentiation, Th1 and Th2 cell differentiation, and the immune‐associated pathways of T cell receptor and B cell receptor signalling pathways in most cancers. It also correlated with genetic markers of immunomodulators in various cancers. LAMP3 and PD‐L1 expression in BRCA and HNSC tissues was higher than that in corresponding adjacent normal tissues by immunohistochemistry. There is a significant correlation between the expression of LAMP3 and PD‐L1.Our study elucidates that LAMP3 has different expression patterns and genetic alteration patterns in different tumours. It is a potential biomarker for immune‐related cancer diagnosis, prognosis and efficacy prediction.

## 1 INTRODUCTION

Approximately 609,820 people in the United States will die from cancer in 2023, equating to 1670 deaths every day.^1^ For men, lung, prostate and colorectal cancer were the most common causes of death, while for women, lung, breast and colorectal cancer were the most common causes of death.^1^ Since 1991, cancer death rates have continued to decline, falling by 33% overall and preventing an estimated 3.8 million deaths from cancer.^1^ This steady progress can be attributed to the decline in smoking; the screening for breast cancer, colorectal cancer and prostate cancer; and the development of adjuvant chemotherapy for colon cancer and breast cancer.^1^ However, the results of traditional therapies are limited, in most cases. There are difficulties with treating complex tumour cells and the complex tumour microenvironment (TME) comprising T cells, macrophages, dendritic cells (DCs) and fibroblasts. Tumour progression and regression are heavily dependent on the dynamic interactions between various cells within the TME.

Cancer immunotherapy, which utilizes the immune system to identify and eradicate cancer, has become a vital part of cancer treatment. Bi‐specific T cell engagers (BiTEs), adoptive cell therapies (ACT), therapeutic cancer vaccines and immune checkpoint blocking are among the most promising treatments.^2^ It may be shocking that cancers with increasing or stable incidence are significantly reduced in mortality by immunotherapy and targeted therapies (leukaemia, melanoma and kidney cancer).^1^ However, many cancer patients who receive the same treatment still do not achieve an objective response. Therefore, by identifying gene expression patterns in pan‐cancer and investigating their relationship with clinical survival and tumour immunity, it is promising to discover new immunotherapeutic targets.

Lysosomes once considered digestive organelles, are now also multifaceted centres of metabolism, nutrient sensing and immune responses.^3^ Not only play lysosomal dysfunction a role in human rare genetic disorders disease, but also includes metabolic diseases, autoimmune diseases and cancer.^4^ Lysosomal‐associated membrane protein 3 (LAMP3), is a member of the lysosome‐associated membrane proteins, which belong to the glycosylated proteins family existing predominantly on the membranes of lysosomes.^5^ It is also known as DC‐LAMP because it was first shown to be gradually induced during the maturation of human DCs and is therefore considered a marker of human mature DCs.^5^ While LAMP1 and LAMP2 are universally studied, little research has been done on LAMP3. It has been shown that LAMP3 overexpression is relevant to unfavourable prognosis in various cancer patients, and LAMP3 may have significance in tumour metastasis and treatment resistance, suggesting that LAMP3 may serve as a molecular marker. Overexpression of LAMP3 promoted invasion and metastasis in cervical cancer and breast cancer.^6^, ^7^ LAMP3 may be associated with metastasis involving signalling downstream of SPP1 in osteosarcoma.^8^ Depletion of LAMP3 suppressed invasion and metastasis through enhancing PKA‐mediated VASP phosphorylation in oesophageal squamous cell carcinoma.^9^ LAMP3 expression was relevant to poorer survival in patients with cervical cancer, head and neck squamous cell carcinoma, stomach cancer and colorectal cancer, while in non‐small cell lung cancer LAMP3 expression was relevant to better survival with patients.^6^, ^10^, ^11^, ^12^, ^13^ In addition, LAMP3 overexpression was relevant to chemotherapy and radiotherapy resistance in breast cancer^14^ and knocking down LAMP3 may promote the sensitivity of patients to cisplatin in prostate cancer.^15^

LAMP3 has yet to have significance in some cancers, and there are also unknown mechanisms that regulate malignant progression of tumours where LAMP3 has been identified. In pan‐cancer, the correlation between LAMP3 and tumour‐infiltrating lymphocytes, immunostimulators, immune inhibitors and so on remains vague. Our study analysed the expression of LAMP3 in pan‐cancer by various databases including TIMER, GTEx, TCGA, GEO, CCLE, HPA, TISIDB and so on. Not only explored the expression and prognosis of LAMP3 in pan‐cancer, but also, we also explored the relationship between LAMP3 expression levels and TIME, such as expression of immunomodulators and immune cell infiltration. This may facilitate comprehension of the significance and potential role of LAMP3 in different tumours.

## 2 MATERIALS AND METHODS

### 2.1 Acquirement of pan‐cancer data and analysis of gene differential expression

The Human Protein Atlas (HPA, https://www.proteinatlas.org/) integrates a variety of omics technologies, such as transcriptomics, antibody‐based imaging, systems biology and mass spectrometry‐based proteomics, to map whole human proteins in organs, tissues and cells. The tissues section of 12 separate sections of the HPA concentrates on the protein and mRNA expression profiles in human tissues of genes. Through antibody‐based protein profiling with conventional and multiplex immunohistochemistry, 44 kinds of normal human tissues were profiled for protein expression. RNA sequencing (RNA‐seq) is used to obtain RNA expression data in 256 various normal tissue types. Two sources of RNA data: Internally generated RNA data from the HPA and RNA‐seq data from the Genotype‐Tissue Expression (GTEx) project. The consensus dataset is based on combined HPA and GTEx.

TIMER (Tumour IMmune Estimation Resource, https://cistrome.shinyapps.io/timer/) is an overall resource to explore systematically immune infiltrates in multiple cancers. With the TIMER algorithm, users can analyse the abundances of B cells, CD4^+^ T cells, CD8^+^ T cells, neutrophils, macrophages and DCs. The differential expression between all the Cancer Genome Atlas (TCGA) tumours and adjacent normal tissues for every gene can be acquired in the section of DiffExp. Box plots are used to visualize gene expression distributions, and the Wilcoxon test is used to explore the importance of multiple expression.

The GEPIA (http://gepia.cancer‐pku.cn/detail.php) is used to explore the TCGA and GTEx RNA‐seq data from 9736 tumours and 8587 normal samples by a standard processing pipeline. The section of boxplot of expression DIY focuses on the RNA expression profiles of LAMP3 in human tumours and normal tissues. The section of stage plot of expression DIY is used to explore the association between the RNA expression profiles of LAMP3 and pathological stage in human different tumours.

UALCAN (https://ualcan.path.uab.edu/index.html) is used to explore cancer OMICS data. Users can easily acquire the publicly OMICS data (CPTAC, MET500, TCGA and CBTTC) in pan‐cancer, recognize biomarkers or ascertain the gene of interest in silico, acquire graphs and plots displaying protein‐coding, miRNA‐coding and lncRNA‐coding gene expression profile and the information of patient survival, study epigenetic regulation of gene expression by promoter methylation, analyse pan‐cancer gene expression and obtain extra information of the genes/targets by linking to other web servers, such as The human protein atlas, HPRD, PubMed, GeneCards, TargetScan, DRUGBANK, Open Targets and the GTEx through this website.

Differential expression of LAMP3 between 17 different cancers and normal samples was studied using the TCGA database in tumour and paired normal tissue. Publicly available R package “ggplot2” and R language software were used to analyse the level of differential expression, which was present in boxplots diagram. All data were preprocessed and normalized by log2 (fpkm+1) transformation.

The 33 kinds of cancers are listed in Table S1 with their full names and abbreviations.

### 2.2 Analysis of the correlation between the expression of LAMP3 and survival

From the TCGA database, the prognosis of patients and clinical phenotyping data were obtained for each sample. The association between LAMP3 expression and prognosis of pan‐cancer patients was investigated using four survival prognosis indices, disease‐free interval (DFI), disease‐specific survival (DSS), overall survival (OS) and progression‐free interval (PFI). R language software and publicly available R package “survival” were employed to conduct a univariate Cox analysis.

The Kaplan–Meier plotter (https://kmplot.com/analysis/) is used to detect the survival in more than 30,000 samples from 21 tumours and the association between the gene expression (mRNA, miRNA and protein).

Not only Cox proportional hazards regression but also false discovery rate computations are used as applied statistical tools. The section of start KM plotter for pan‐cancer focuses on the relapse‐free survival (RFS) and OS to explore the expression of LAMP3 in various tumours from RNA‐seq data.

### 2.3 Genetic alteration analysis of LAMP3

The genetic alteration of genes is analysed in online cBioPortal (https://www.cbioportal.org/), which including 10,967 samples including 10,953 patients from the TCGA database in pan‐cancer. The sections of mutations, cancer types summary and plots are used to explore the genomic mutation information and somatic mutation frequency of LAMP3.

### 2.4 Analysis of the DNA methylation and copy number of LAMP3

The correlation between DNA methylation levels, copy number variations (CNVs) and gene expression using the Hmisc package from the TCGA database. The Pearson correlation coefficient (*r*) measures the linear relationship between two continuous variables. A positive correlation suggests that higher methylation levels are associated with higher gene expression, while a negative correlation indicates an inverse relationship.

### 2.5 Analysis of the correlation between LAMP3 and immune cell infiltration, immune modulator genes

Users can explore the association of any gene expression with immune infiltration level and compare the difference of the expression of given gene with immune infiltration level with multiple somatic copy number alterations in various tumours in the section of gene and SCNA of TIMER, respectively. According to GISTIC 2.0, SCNAs include high amplifications, deep deletions, arm‐level deletions arm‐level gains and diploid/normal. The box plots were used to detect the distributions of each immune subset at each copy number status in cancer. A two‐sided Wilcoxon rank‐sum test was used to analyse the infiltration level difference between SCNA category and the normal.

TISIDB (http://cis.hku.hk/TISIDB/) can be used to explore different kinds of heterogeneous data tumour and immune system interaction. It involves literature mining results from PubMed database, detecting sensitivity and resistance of tumour cells to T cell‐mediated killing from high throughput screening data, patient cohorts with immunotherapy from exome and RNA‐seq data, genomics, transcriptomic and clinical data of 30 tumours from the TCGA database, DrugBank, UniProt, GO, etc.

The section of lymphocytes including immunologically relevant features of 28 kinds of (tumour‐infiltrating lymphocyte) TIL from Charoentong's study is used to analyse the correlation between the abundance of TILs and the expression of LAMP3. On the basis of gene expression profiles, the relative abundance of TILs was inferred for every tumour by using gene set variation analysis (GSVA). The sections of immunomodulator and chemokine are used to explore the relations between immunomodulators, chemokines (or receptors) and expression of LAMP3, respectively.

### 2.6 Functional enrichment analysis of LAMP3

Firstly, we collected gene sets with a greater than 0.3 absolute value of correlation with LAMP3 in each tumour in the UALCAN database. KEGG analysis of the gene sets was executed via the DAVID database (https://david.ncifcrf.gov/home.jsp), which can unravel the biological functions of large lists of genes.

### 2.7 Immunohistochemistry (IHC)

Twelve BRCA patients' samples and 10 adjacent non‐cancerous tissues, and 10 HNSC patients' samples and 10 adjacent non‐cancerous tissues were from the Shanxi Cancer Hospital. Our study was permitted by the Ethics Committee of Shanxi Medical University (Approval No. 2021SLL047). The LAMP3 and PD‐L1 primary antibodies were obtained from proteintech. The specific steps of Immunohistochemistry have been described before.^16^ The stained specimens were scanned with an automatic digital section scanner (Aperio Image Scope).

### 2.8 Immunotherapy prediction analysis

The Spearman correlation analysis was utilized to calculate the statistical correlations between LAMP3 and PD‐L1. Two treatment cohorts with immune checkpoint blockade (ICB) to analyse the predictive power of LAMP3 for immunotherapy response, the GSE93157 HNSC cohort and PRJEB23709 melanoma cohort from Tumour Immunotherapy Gene Expression Resource (TIGER) database.

### 2.9 Statistical analysis

Students' *t*‐test and Wilcoxon rank sum test were used to compare differences between the two experimental groups. One‐way analysis of variance (anova) was used to compare more than two experimental groups. *p* < 0.05 was considered statistically significant. * *p* < 0.05; ** *p* < 0.01; *** *p* < 0.001. “ns” indicated no significance.

## 3 RESULTS

### 3.1 The Landscape expression and pan‐cancer expression of LAMP3

LAMP3 is primarily expressed in many human tissues and organs, especially in the respiratory system, bone marrow and lymphoid tissues in the HPA (Figure S1A). The RNA of LAMP3 is mainly expressed in the lung, appendix, thymus, salivary gland, testis, tonsil, lymph node, urinary bladder and spleen from the consensus dataset including the HPA and the GTEx database (Figure S1B). The protein of LAMP3 is primarily expressed in lung, lymph node, tonsil from HPA database including 144 samples (Figure S1C).

**FIGURE 1:**
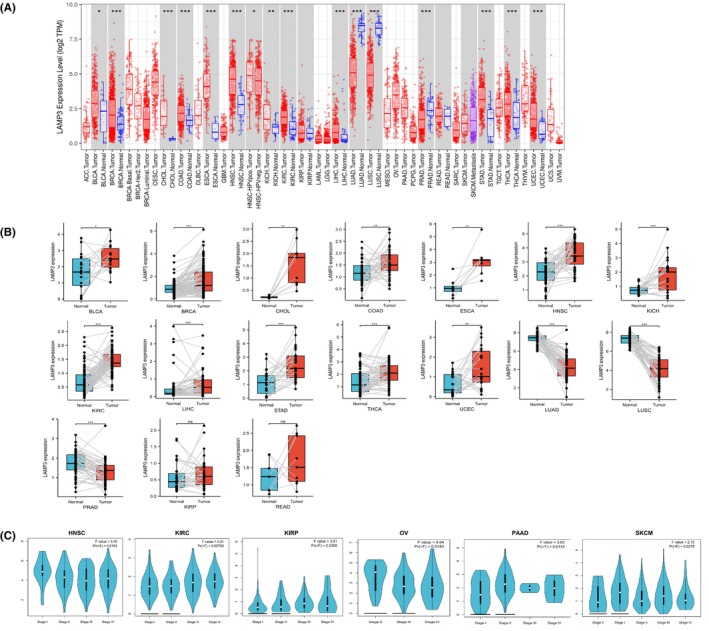
RNA expression profile of LAMP3 in pan‐cancer and the correlation between the LAMP3 expression and the pathological stage of pan‐cancer. (A) The RNA expression of LAMP3 in various cancers and normal tissues from the TCGA data in TIMER. (B) Paired differential analysis of LAMP3 expression in matched tumour and normal samples from the TCGA data. (C) Correlations between the LAMP3 expression and the pathological stage of tumours from GEPIA. Log2 (TPM + 1) was used for log scale.

The 33 kinds of cancers are listed in Table S1 with their full names and abbreviations. Our analysis of LAMP3 expression in various cancers in TIMER from the TCGA database elucidated that LAMP3 was observably upregulated in 13 cancers, including BLCA, BRCA, CHOL, COAD, ESCA, HNSC, KICH, KIRC, LIHC, STAD, THCA and UCEC (all *p* < 0.05) (Figure 1A). LAMP3 was markedly downregulated in LUAD, LUSC and PRAD (all *p* < 0.05) (Figure 1A). In GEPIA, combined data from TCGA and GTEx revealed that LAMP3 expression increased in BLCA, BRCA, CESC, COAD, DLBC, ESCA, HNSC, OV, PAAD, READ, STAD, THCA, THYM, UCEC, UCS and LAMP3 was significantly downregulated in LUAD, LUSC and TGCT (Figure S1D). TCGA data in UALCAN indicated that LAMP3 expression increased in BLCA, BRCA, CESC, CHOL, COAD, ESCA, GBM, HNSC, KICH, KIRC, LIHC, PCPG, SARC, THCA, STAD, UCEC and LAMP3 was downregulated in LUAD, LUSC, PAAD, PRAD and THYM (Figure S1E).

In addition, Student's *t*‐test was used to investigate paired differential expression demonstrating that the mRNA expression of LAMP3 was enhanced in BLCA, BRCA, CHOL, COAD, ESCA, HNSC, KICH, KIRC, LIHC, STAD, THCA and UCEC. The mRNA expression of LAMP3 was decreased in LUAD, LUSC and PRAD (all *p* < 0.05) (Figure 1B). However, there was no statistical difference in LAMP3 expression between KIRP, READ and corresponding normal tissues. The mRNA expression of LAMP3 in other tumours could not be explored because of the absence of adequate paired samples.

### 3.2 Analysis of the relationship between the LAMP3 expression and pan‐cancer pathological stage

Next, we explored the correlation between LAMP3 expression and pathological stage in the GEPIA database. Interestingly, our results demonstrated that LAMP3 expression was markedly related to tumour stage in HNSC, KIRC, KIRP, OV, PAAD and SKCM (all *p* < 0.05) (Figure 1C), but in other tumours, it did not correlate with the pathological stage (*p* > 0.05) (Figure S2A–R). Overall, these results supported the notion that LAMP3 expression had a guiding significance for the pathological staging of HNSC, KIRC, KIRP, OV, PAAD and SKCM.

### 3.3 Pan‐cancer prognostic value of LAMP3

To clarify whether the expression of LAMP3 is related to the prognostic of tumours, the Kaplan–Meier analysis was performed for OS, DSS, DFI and PFI via forest plots for pan‐cancer. Our results elucidated that LAMP3 was a crucial risk factor for OS in KIRC, KIRP, LGG, PAAD, THYM and UCEC by the univariate Cox regression (all *p* < 0.05), while a protective factor in BRCA, LUAD, MESO, OV and SKCM (all *p* < 0.05) (Figure S3A). Regarding DSS of the 32 cancers, LAMP3 was a prominent risk factor in DLBC, GBM, KICH, KIRC, KIRP, LGG, PAAD, TGCT and UCEC but played a protective role for OV and SKCM (all *p* < 0.05) (Figure S3B). In addition, our results elucidated that LAMP3 was a remarkable risk factor for DFI in KIRP and THCA, and it was a marked risk factor for PFI in GBM, KICH, KIRC, KIRP, LGG, PAAD and UCEC, while a favourable factor in BRCA, MESO and SKCM (all *p* < 0.05) (Figure S3C,D).

**FIGURE 2:**
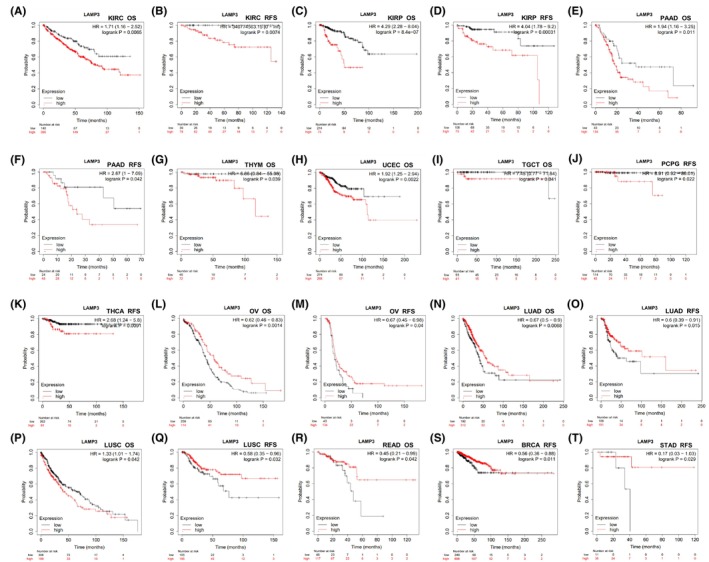
Survival analyses of LAMP3 expression in pan‐cancer in Kaplan–Meier plotter. OS of (A) KIRC, (C) KIRP, (E) PAAD, (G) THYM, (H) UCEC, (I) TGCT, (L) OV, (N) LUAD, (P) LUSC, (R) READ, RFS of (B) KIRC, (D) KIRP, (F) PAAD, (J) PCPG, (K) THCA, (M) OV, (O) LUAD, (Q) LUSC, (S) BRCA, (T) STAD.

Subsequently, the prognostic value of LAMP3 expression in pan‐cancer was assessed in online Kaplan–Meier Plotter web (Table S2), whose data mostly come from TCGA. LAMP3 expression was related to unfavourable prognosis in patients with KIRC, KIRP and PAAD as measured by Kaplan–Meier survival curves for both OS and RFS (all *p* < 0.05) (Figure 2A–F). High expression of LAMP3 for OS was related to poor prognosis of patients in THYM, UCEC and TGCT (all *p* < 0.05) (Figure 2G–I). High expression of LAMP3 for RFS was related to poor prognosis of patients with PCPG and THCA (all *p* < 0.05) (Figure 2J,K). However, high expression of LAMP3 has a sharp association with longer survival time in patients with OV, LUAD and LUSC for both OS and RFS (all *p* < 0.05) (Figure 2L–Q). Interestingly, high expression of LAMP3 has a marked correlation with beneficial prognosis of READ patients for OS, and it has a marked correlation with good survival outcomes in patients with BRCA and STAD (all *p* < 0.05) (Figure 2R–T).

Subsequently, we analysed the role of LAMP3 in various cancers in GEPIA (Table S3), whose data mostly come from RNA‐seq data from TCGA. Our results elucidated that high expression of LAMP3 has an obvious relationship with worse OS in LGG and UVM, DFS in GBM, KICH and KIRC, both OS and DFS in KIRP (Figure S4A–G) while it was positively correlated with patients of SKCM for both OS and DFS, OS in BRCA and OV, DFS in CHOL and UCS (all *p* < 0.05) (Figure S4H–M).

### 3.4 Genetic alteration, methylation and copy number level of LAMP3 in pan‐cancer

**FIGURE 3:**
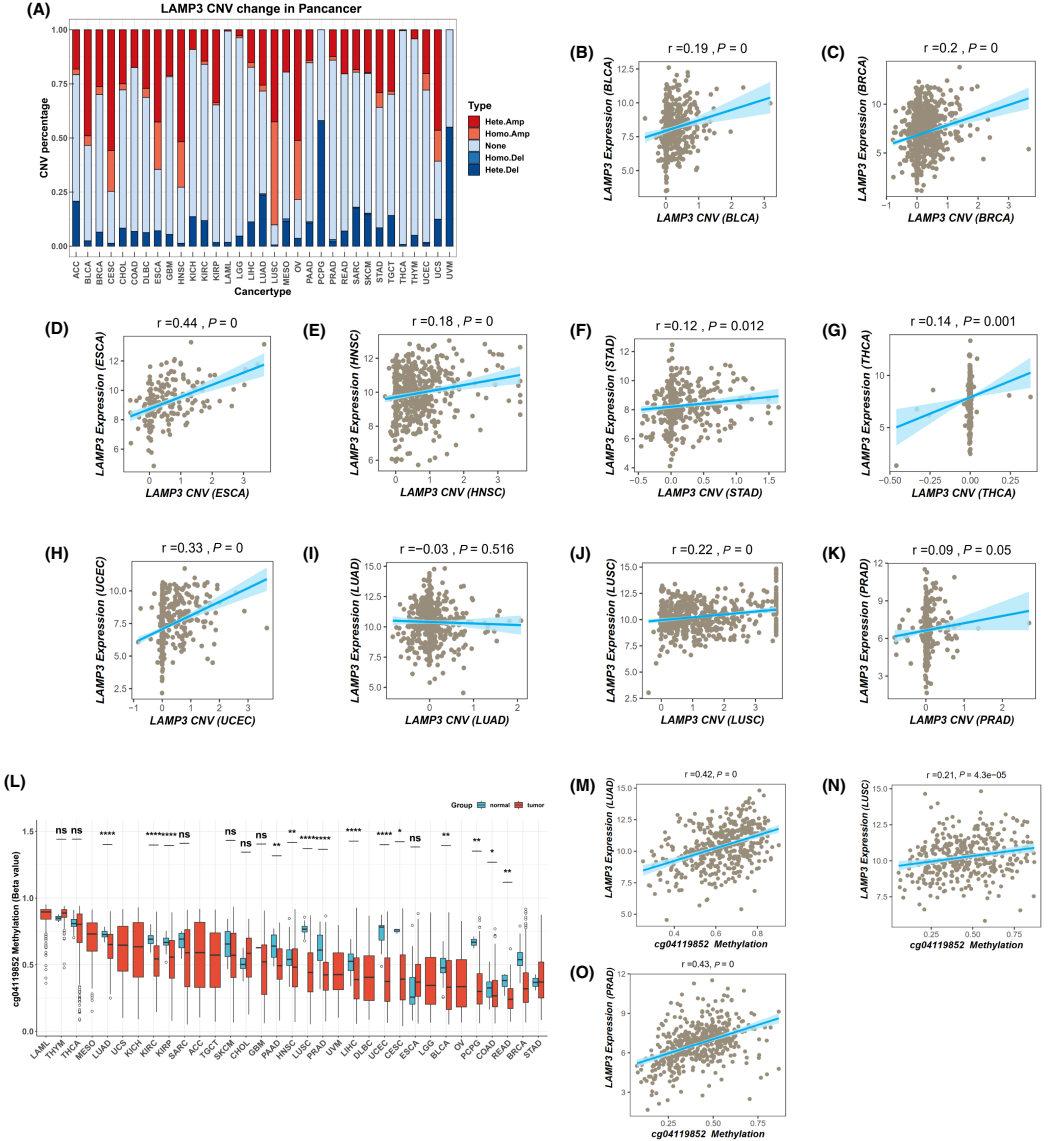
Analysis of the copy number and the methylation and of LAMP3 in pan‐cancer based on TCGA database. (A) Copy number of LAMP3 in pan‐cancer. (B–K) The spot correlation plot between the copy number and the LAMP3 expression in pan‐cancer. (L) Methylation of LAMP3 in pan‐cancer. (M–O) The spot correlation plot between the methylation and the LAMP3 expression in LUAD (M), LUSC (N) and PRAD (O).

LAMP3 gene mutations in pan‐cancer were explored by the cBioPortal online tool, in which all TCGA Pan‐cancer data. Ninety‐nine mutation sites were found between amino acids 0 and 416, including 77 missense mutations, 18 truncations, 2 splicing and 2 fusions, with K5Nfs*15 being the most common mutation site (Figure S5A). There were three types of genetic alterations including amplification, missense mutation and deep deletion. Most LAMP3 genetic alterations were mainly detected in NSCLC, OV, HNSC, CESC, ESCA and endometrial cancer (Figure S5B). Amplification of LAMP3 mRNA was found in many tumours including BLCA, BRCA, CESC, endometrial cancer, ESCA, HNSC, NSCLC and OV (Figure S5C). In addition, we investigated the correlation between the LAMP3 expression and gene mutation from the TIMER database. The result showed that there was no correlation between LAMP3 expression and its mutation in pan‐cancer (*P* > 0.05) (Figure S6). We also analysed the correlation between the LAMP3 expression and copy number from the TCGA database in pan‐cancer. Our results revealed that in BLCA, BRCA, ESCA, HNSC, STAD, THCA and UCEC with high LAMP3 expression, the expression of LAMP3 was positively correlated with the copy number (*p* < 0.05) (Figure 3A–H). There was no correlation between LAMP3 expression and copy number in tumours (LUAD and PRAD) with low LAMP3 expression (*p* > 0.05) (Figures 3I,K). The expression of LAMP3 was positively correlated with the copy number in LUSC with low LAMP3 expression (*p* < 0.05) (Figure 3J). The above results indicated that LAMP3 expression in most tumours was related to copy number.

Aberrant DNA methylation is strongly associated with all kinds of diseases such as cancer.^17^ Our results showed that the beta value of cg04119852 methylation site was remarkably decreased in BLCA, COAD, HNSC, KIRC, LIHC and UCEC with high LAMP3 expression. LUAD, LUSC and PRAD with low LAMP3 expression had a lower level of LAMP3 methylation with 04119852 site, and the expression of LAMP3 was positively correlated with the methylation (*p* < 0.05) (Figure 3L–O).

### 3.5 Analysis of LAMP3 expression and immune cell infiltration in pan‐cancer

The TME contains several non‐redundant features that promote immunosuppression and inhibit anticancer immune responses, such as physical barriers, recruiting the upregulation of ligands on tumour cells and suppressive immune cells binding to immunoinhibitory receptors. We investigated the relationship between LAMP3 expression and immune infiltration of six types of cells (B cells, CD8^+^T cells, CD4^+^T cells, macrophages, neutrophils and DCs) from the TIMER database. In 31 out of 39 tumours, LAMP3 has a sharp association with tumour purity. At the same time, the results showed that 30 tumours were related to B cells, 30 tumours were related to CD8^+^T cells, 31 tumours were related to CD4^+^T cells, 24 tumours were related to macrophages, 33 tumours were related to neutrophils and 37 tumours were related to DCs (Table S4). Most remarkable, LAMP3 was related to all 6 types of cells in 17 out of 39 cancer types, namely BRCA‐luminal, COAD, HNSC, HNSC‐HPVneg, KIRC, KIRP, LGG, LIHC, LUAD, LUSC, PAAD, PRAD, SARC, SKCM, SKCM‐metastasis, STAD and THCA (Table S4). On the contrary, LAMP3 was almost irrelevant with all 6 types of cells in 4 out of 39 cancer types, namely ACC, DLBC, KICH and UVM (Table S4).

Next, BRCA, OV, KIRC, PAAD, LUSC and LUAD were selected and divided into three categories to detect whether the expression of LAMP3 is related to immune infiltration. BRCA and OV represent cancers in which patients with high LAMP3 expression had beneficial survival, while KIRC and PAAD represent cancers in which patients with high LAMP3 expression had unfavourable survival. LUSC and LUAD represent cancers in which patients with low LAMP3 expression had beneficial survival. In addition, LAMP3 has a negative association with tumour purity in all three types of tumours, and it has a positive association with the infiltration levels of 6 types of immune cells in all three types of tumours (Figure S7A–C).

In a word, the above results indicate that LAMP3 expression is significantly related to immune infiltration in the TME in many kinds of cancers, while Cancer patients' survival may not be affected by LAMP3 expression by influencing immune infiltration.

### 3.6 Analysis of the relationship between LAMP3 expression and immunogenetics in pan‐cancer

**FIGURE 4:**
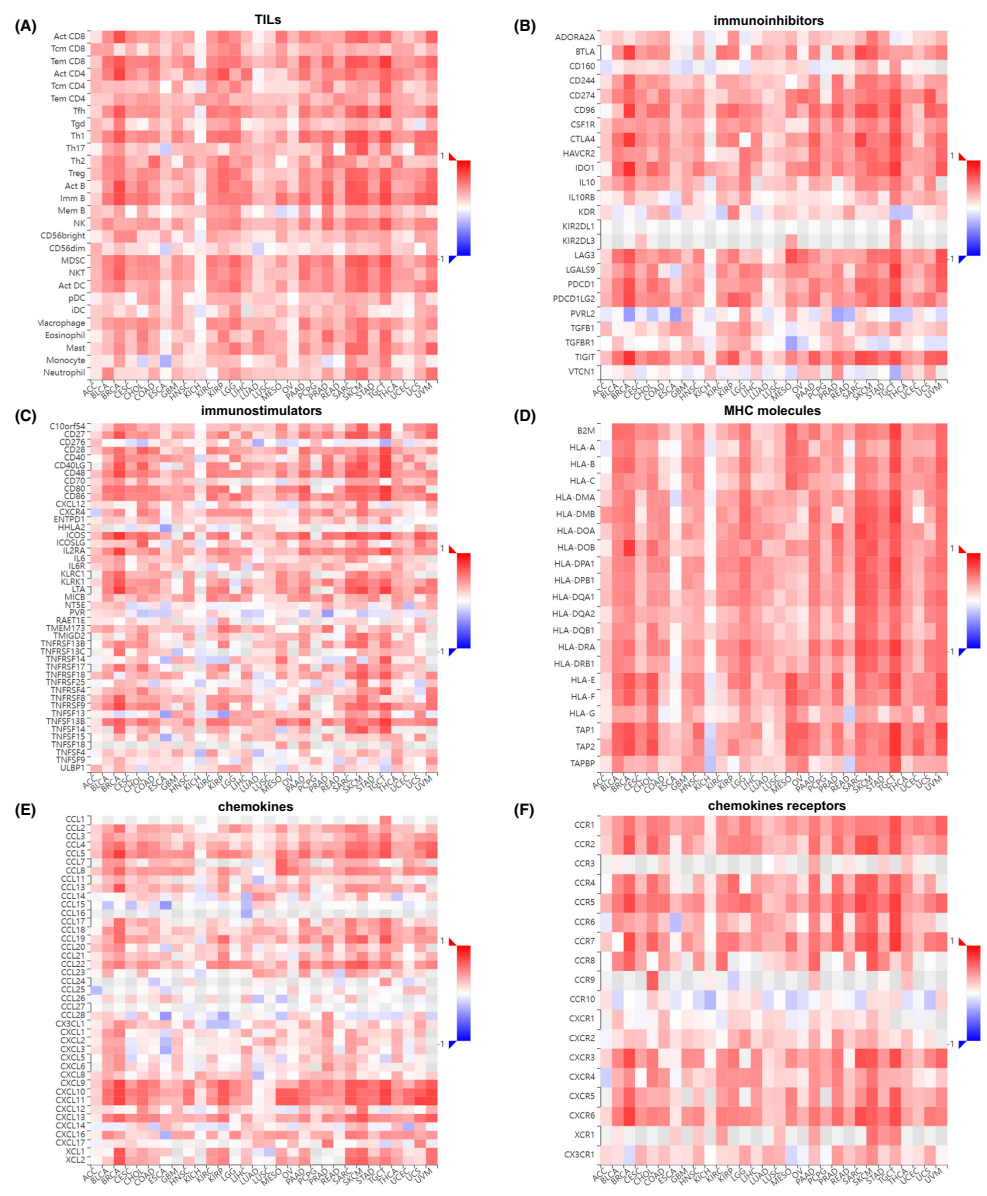
Heatmap analysis of the correlation between LAMP3 and (A) TILs, (B) immunoinhibitors, (C) immunostimulators, (D) MHC molecules, (E) chemokines and (F) chemokines receptors in tumours.

To further understand the correlation between LAMP3 expression and immune regulation, we explored heat maps of LAMP3 with immunoinhibitors, immunostimulators, TILs, major histocompatibility complex (MHC) molecules, chemokines and chemokines receptors from TISIDB database. Our results demonstrated that LAMP3 displayed a significantly positive relationship with nearly all TILs in various cancers except KICH (Figure 4A). Furthermore, both immunoinhibitors and immunostimulators were sharply associated with LAMP3 in pan‐cancer expect KICH (Figure 4B,C). Among immunoinhibitors, PVRL2, CD160, KDR and VTCN1 were also negatively correlated with LAMP3 expression in many tumours (Figure 4B). Among immunostimulators, CD276, PVR, TNFRSF14, TNFRSF25 and TNFSF9 were also negatively correlated with LAMP3 expression in some tumours (Figure 4C). Similarly, LAMP3 showed a significantly positive relationship with nearly all MHC molecules in various cancers except KICH (Figure 4D). In most tumours, both chemokines and chemokines receptors were heavily associated with LAMP3 in pan‐cancer except KICH (Figure 4E,F). Among chemokines, especially CCL4, CCL5, CCL8, CXCL9, CXCL10 and CXCL11 were markedly positive correlated with LAMP3 expression in most cancers (Figure 4E). Among chemokines receptors, especially CCR1, CCR2, CCR4, CCR5, CCR7, CXCR3, CXCR4, CXCR5 and CXCR6 were significantly positive correlated with LAMP3 expression in almost all cancers (Figure 4F).

To sum up, it appears that LAMP3 may regulate immune‐related functions in the tumour immune microenvironment except KICH.

### 3.7 Analysis of the relationship between LAMP3 copy number variations, LAMP3 mutations and immune infiltrates in pan‐cancer

From the previous results, LAMP3 genetic alterations were found in a variety of cancers (Figure S5A–C). Then we analysed that LAMP3 copy number variations were significantly associated with immune infiltrates mainly in ten kinds of cancer, namely OV, PAAD, SKCM, UCEC, TCGT, HNSC, HNSC‐HPVneg, LUAD, LUSC and STAD (Figure S8A–J). High amplification of LAMP3 was associated with immune infiltrates cells in HNSC, HNSC‐HPVneg, LUSC, OV, STAD and UCEC (Figure S8A,B,D,E,H,J). However, deep deletion of LAMP3 was associated with immune infiltrate cells in LUAD and SKCM (Figure S8C,G).

Then we analysed the difference between wild‐type and mutated LAMP3 regarding their immune‐regulating functions from TIMER database. Mutant LAMP3 had a lower immune infiltration level of B cells than wild‐type LAMP3 in LUAD. However, mutant LAMP3 had a higher immune infiltration level of B cells than wild‐type LAMP3 in UCEC. Mutant LAMP3 had a higher immune infiltration level of myeloid dendritic cells and CD8 + T cells than wild‐type LAMP3 in BRCA and UCEC. Mutant LAMP3 also had a higher immune infiltration level of neutrophils in COAD. Mutant LAMP3 had a lower immune infiltration level of myeloid dendritic cells than wild‐type LAMP3 in LIHC (Figure S9). The above results show that LAMP3 mutation could affect immune infiltration across multiple cancer types and immune cell types.

In conclusion, LAMP3 copy number variations and mutations have a sharp correlation with immune infiltrates in pan‐cancer.

### 3.8 Enrichment analysis of LAMP3 in pan‐cancer

**FIGURE 5:**
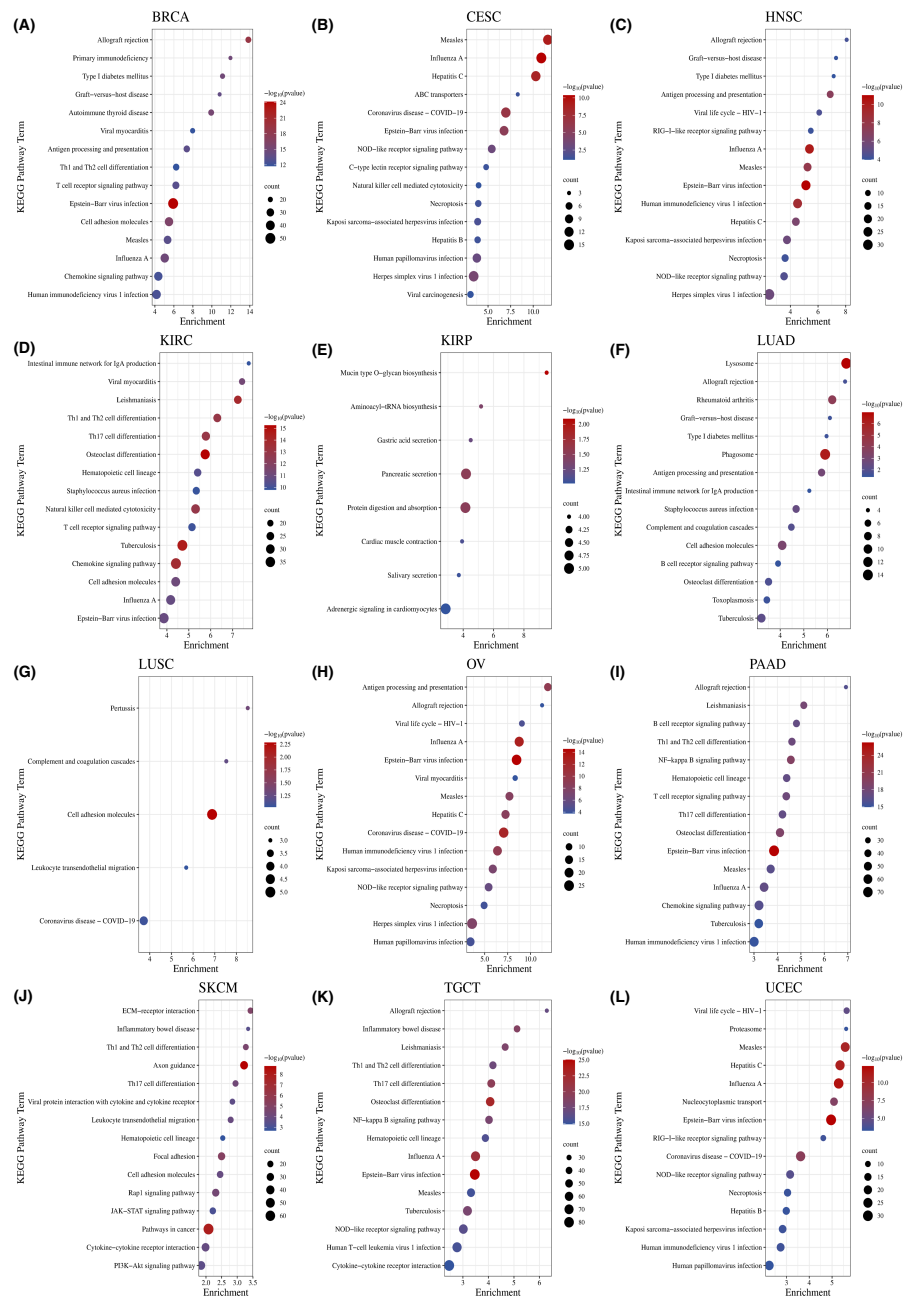
KEGG annotations of LAMP3 indicated 12 types of tumours. The KEGG bubble plots of (A) BRCA, (B) CESC, (C) HNSC, (D) KIRC, (E) KIRP, (F) LUAD, (G) LUSC, (H) OV, (I) PAAD, (J) SKCM, (K) TGCT, (L) UCEC.

To clarify the underlying biological function of LAMP3 in tumours, we performed Kyoto Encyclopedia of Genes and Genomes (KEGG) by gene sets with a greater than 0.3 absolute value of correlation with LAMP3 in each tumour in the UALCAN database. We chose the top 10 most meaningful KEGG results to make bubble maps in pan‐cancer except KIRP and LUSC, whose KEGG results have only eight and five kinds, respectively. The KEGG‐enriched terms revealed that LAMP3 was critically associated with immune‐associated tumorigenic virus infectious diseases, including Epstein–Barr virus infection, influenza A, measles, hepatitis B, hepatitis C, coronavirus disease‐COVID‐19, human immunodeficiency virus 1 infection, herpes simplex virus 1 infection and so on in pan‐cancer expect KIRP (Figure 5A–L). Meanwhile, the major associations of LAMP3 with pan‐cancer existed in the immune‐associated processes of Antigen processing and presentation, Th17 cell differentiation, Th1 and Th2 cell differentiation, and the immune‐associated pathways of T cell receptor and B cell receptor signalling pathway. Meanwhile, LAMP3 was associated with biological processes such as necroptosis in CESC, OV, UCEC, cell adhesion in BRCA, KIRC, LUAD, LUSC, SKCM and type I diabetes mellitus in BRCA, HNSC and LUAD. It is worth mentioning that the most relevant pathway for LAMP3 in pan‐cancer is NOD‐like receptor signalling pathway.

Surprisingly, the major associations of LAMP3 with KIRP existed in the processes of mucin type O‐glycan biosynthesis, secretion, protein digestion and absorption, aminoacyl‐tRNA biosynthesis and so on, not immune‐associated processes. As a result of these findings, it was concluded that LAMP3 was significantly associated with tumour progression, especially within the immune microenvironment.

### 3.9 Experimental validation and the predictive role of LAMP3 in cancer immunotherapy

**FIGURE 6:**
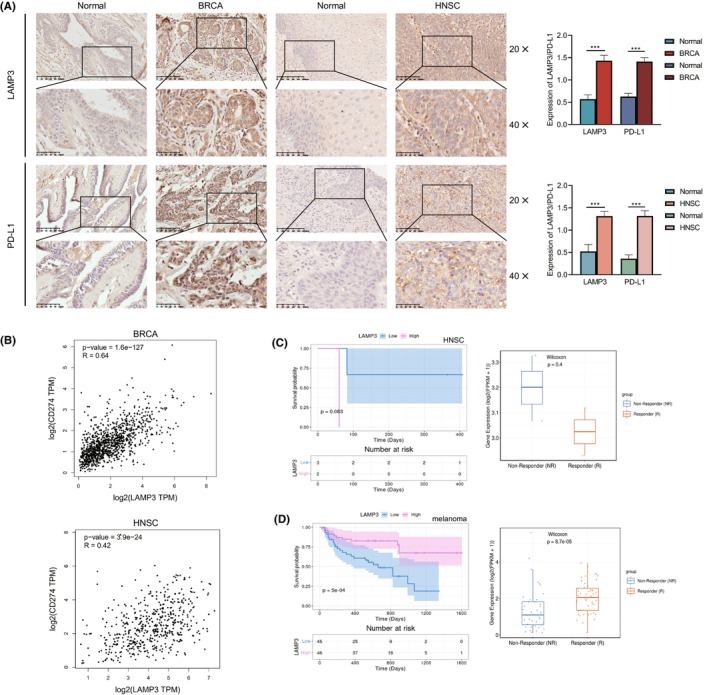
LAMP3 predicts the immunotherapy response of cancer patients. (A) Immunohistochemistry staining images and scores of LAMP3 and PD‐L1 in BRCA, HNSC and corresponding normal tissues. (B) The correlation analysis between LAMP3 and PD‐L1 in BRCA and HNSC from GEPIA. (C) Survival curve for low‐LAMP3 and high‐LAMP3 patient groups in GSE93157 cohort in TIGER (anti‐PD‐1 and HNSC). (D) Survival curve for low‐LAMP3 and high‐LAMP3 patient groups in PRJEB23709 cohort in TIGER (anti‐PD‐1/anti‐CTLA‐4 combined therapy and melanoma).

Immunotherapies, particularly immune checkpoint inhibitors (ICIs), such as the anti‐PD‐L1, anti‐PD‐1 and anti‐CTLA4, have revolutionized the treatment of cancer.^18^ To validate the expression of LAMP3 in human tumour tissues and its relationship with immune checkpoint proteins, we detected the correlation between LAMP3 and immune protein PD‐L1 in clinic samples by immunohistochemistry. The results showed that LAMP3 and PD‐L1 expression in BRCA and HNSC tissues was higher than that in corresponding adjacent normal tissues (Figure 6A). According to the IHC staining score, our statistical analysis results showed that the *P* value is statistically significant (*p* < 0.05) (Figure 6A). The RNA expression of LAMP3 correlated with PD‐L1 (*p* < 0.05) by Spearman analysis in GEPIA (Figure 6B).

Subsequently, we analysed the predictive role of LAMP3 in patients with cancer who received immune therapies from the TIGER database. The relationship between LAMP3 and the response to anti‐PD‐1 treatment in HNSC patients showed that the survival of patients with high LAMP3 expression was worse than those with low LAMP3 expression (*p* = 0.083) (Figure 6C). The immunotherapeutic response in a cohort of GSE93157, the response group proved the lower LAMP3 expression level in LAMP3 with pembrolizumab or nivolumab in HNSC (*p* = 0.4) (Figure 6C). It is worth mentioning that *p* > 0.05 may be due to the small number of anti‐PD‐1 therapy HNSC cases.

The relationship between LAMP3 and the response to anti‐PD‐1 monotherapy and anti‐PD‐1/anti‐CTLA‐4 combined therapy in melanoma patients showed that the survival of patients with high LAMP3 expression was better than those with low LAMP3 expression (*p* < 0.05) (Figure 6D). The immunotherapeutic response in a cohort of PRJEB23709, the response group proved the higher LAMP3 expression level in LAMP3 with anti‐PD‐1 monotherapy and anti‐PD‐1/anti‐CTLA‐4 combined therapy in melanoma (*p* < 0.05) (Figure 6D).

## 4 DISCUSSION

Lysosomes are involved with following processes: recycling of cellular waste, cellular signalling, degradation and metabolism.^19^ A growing body of evidence also hints at the role of abnormal lysosomal proteins, which lead to disrupted lysosomal dysfunction in inflammatory and autoimmune diseases, metabolic disorders, cancer and so on.^20^ LAMPs (LAMP1, LAMP2, LAMP3, LAMP4 and LAMP5) exist primarily on lysosomal membranes, which are related to cell biology and several cellular processes such as autophagy and ageing.^21^ Among them, LAMP1, LAMP2, LAMP4 and LAMP5 have been studied in relation to cancer and immunity.^22^, ^23^, ^24^ LAMP1 expression on the cell surface is commonly found also in some types of immune cells, such as natural killer cells (NK cells) and T cells and is commonly used as a marker for degranulation and active cytotoxicity.^5^ In patients with ANCA‐negative piFNGN, autoantibodies to LAMP2 could bind native glomerular but not neutrophil LAMP‐2, suggesting a role in pathogenesis.^25^ Studies have shown that LAMP4 was associated with the antigen presentation process.^5^ LAMP5 limits type 1 interferon responses by sorting TLR9 to late endosomal compartments.^26^ Our study detected that LAMP3 is mainly expressed in Respiratory system, Bone marrow and Lymphoid tissues. Although LAMP3 is not ubiquitously expressed and its function in some tumours has been reported, the correlation between LAMP3 and pan‐cancer remains to be systematically studied.

Our analyses showed that LAMP3 expression is high in most tumours and low in LUAD, LUSC and PRAD. Gene mutation, copy number variation (CNV) and DNA methylation are the main forms of gene expression regulation. Our results showed that there was no correlation between LAMP3 expression and its mutation in pan‐cancer. LUAD, LUSC and PRAD had a lower level of LAMP3 methylation with the cg04119852 site, and the expression of LAMP3 was positively correlated with the methylation. This may be the reason for the low expression of LAMP3 in LUAD, LUSC and PRAD. In addition, in most tumours with high LAMP3 expression, the expression of LAMP3 was positively correlated with the copy number. There was no correlation between LAMP3 expression and copy number in tumours (LUAD and PRAD) with low LAMP3 expression. It was worth watching that in LUSC with low LAMP3 expression, the expression of LAMP3 was positively correlated with the copy number. The above results indicated that LAMP3 expression was more related to its methylation than copy number.

Previous reports showed that LAMP3 was expressed which depends on oxygen concentration and it contributed to tamoxifen resistance in breast cancer.^14^, ^27^ LAMP3 overexpression promoted the metastasis in cervical cancer, and it may be a novel predictor of both gastric cancer and cervical cancer.^6^, ^11^ These studies are consistent with our results. Besides the tumours analysed in the database, there is a report that showed that LAMP3 promoted Osteosarcoma cell proliferation by regulating TP53 expression.^28^ Our study indicated that LAMP3 was significantly associated with a stage in several tumours, which has a relationship with the prognosis of patients. The lower the overall stage value, the more early the tumour is and the better the prognosis is generally. A higher overall stage indicates that the tumour is at a more advanced stage, the treatment is more complex and the prognosis is worse. Our results showed that the scores of OV and HNSC stages 1–4 were lower and lower, and the larger the stage, the lower the LAMP3 expression, elucidating that the higher the LAMP3 expression in the two tumours, the better the prognosis. While the higher the LAMP3 expression in KIRC, KIRP, PAAD and SKCM, the worse the prognosis.

Then, to test our hypothesis, we analysed the correlation between LAMP3 expression and the prognosis of various tumours. Patients with tumours with high LAMP3 expression showed a poor prognosis, such as KIRC and KIRP, while patients with tumours with high LAMP3 expression showed a good prognosis such as BRCA and OV for the univariate Cox regression analysis, and survival analysis in online Kaplan–Meier and GEPIA. These results explain the relationship between LAMP3 expression and staging in OV. High LAMP3 expression was obviously related to poor prognosis of patients in KIRC, KIRP, LGG, PAAD, THYM, UCEC, GBM, KICH, TGCT and THCA and it was related to good prognosis of patients in BRCA, LUAD, OV and SKCM for survival analysis either the univariate Cox regression analysis, online Kaplan–Meier or GEPIA. We classified the above conditions into three types: high LAMP3 expression and poor prognosis (e.g. KIRC, KIRP, LGG, PAAD, THYM, UCEC, GBM, KICH, TGCT and THCA); low LAMP3 expression and good prognosis (e.g. LUAD); high LAMP3 expression and good prognosis (e.g. BRCA and OV). This results that the higher the LAMP3 expression, the better prognosis does not conform to conventional thinking. In OV, the high expression group of tertiary lymphoid structures (TLSs) including the LAMP3 gene exhibited a favourable prognosis for OS and this result is consistent with our database analysis.^29^ LAMP3 was reported to be sharply related to locoregional control in patients who received radiation therapy.^27^ Only in breast cancer patients undergoing lumpectomy and radiation therapy did those with high expression of LAMP3 have worse DFS and OS than those with low levels.^27^ Therefore, the report does not invalidate the results of high LAMP3 expression and favourable prognosis in BRCA from our database analysis. Since both OV and BRCA are hormone‐related tumours, their high expression and good prognosis may be related to hormones, but this needs to be proven experimentally. In addition, it may be related to the immune effect of LAMP3. For example, the high expression of CXCL11 was related to a good prognosis in COAD.^30^ The authors found that antitumor immune cells were more prevalent in the group with high CXCL11 expression, and protumor immune cells were less prevalent. In addition, the expression of CXCL11 has a positive correlation with immunosuppressive molecules such as PD‐L1, and it was taken as an independent prognostic marker in patients with COAD.^30^ In Figure 4B, our results showed that LAMP3 has a positive correlation with immunoinhibitors, including PD‐L1 in BRCA and OV. So, LAMP3 may promote antitumor immunity to benefit survival.

LAMP3 is identified as DC‐LAMP, which is induced and is considered as a marker in human mature Dcs.^31^ LAMP3 was identified as a potential biomarker of immunotherapy, and it was related to the prognosis of LUAD patients.^32^ Single‐cell transcriptome and TCR sequencing on CD45^+^ immune cells from 12 surgical resected IIIA NSCLC patients showed the number of LAMP3^+^ DCs increased.^33^ A cluster of LAMP3^+^ DCs could migrate from hepatocellular carcinoma to LNs.^34^ Although we have a certain understanding of the relationship between LAMP3 and immunity, we still lack systematic study on the relationship between LAMP3 and immunity in pan‐cancer. Tumour infiltrating immune cells can both promote and inhibit the progression of tumours.^35^ Our study demonstrated that in most cancers LAMP3 expression was related to 6 kinds of immune cell infiltration, which was related to unfavourable prognosis and chemotherapy resistance.^36^ In breast cancer, LAMP3 was associated with tamoxifen resistance, radioresistance and inflammation,^14^, ^37^, ^38^ and its downregulation promoted the sensitivity of cisplatin in prostate cancer.^15^ Interestingly, in GBM, LAMP3 expression was negatively correlated with all six kinds of immune cell infiltration. In THYM and UVM, LAMP3 was negatively associated with five and four types of immune cell infiltration, respectively. Therefore, LAMP3 may interact with immune cells in many tumours and its expression was widely related to multiple immune factors and immune cell invasion of tumours. Furthermore, we found that the expression of LAMP3 was sharply related to copy number variants and immune infiltration in pan‐cancer. It was suggested that LAMP3 expression and immune infiltration were correlated, but various tumour types exhibited differences and it may have a positive promoting effect on patient survival by affecting immune infiltration in TME. We also showed that LAMP3 mutation could affect immune infiltration across multiple cancer types and immune cell types.

According to KEGG enrichment analyses, LAMP3 may function as a regulator of immune‐associated processes antigen processing and presentation, Th1 and Th2 cell differentiation, Th17 cell differentiation and immune‐associated pathways. Meanwhile, LAMP3 was related to biological processes including necroptosis, cell adhesion, type I diabetes mellitus and NOD‐like receptor signalling pathway. NOD‐like receptor signalling pathways have a crucial role in inflammation‐associated tumorigenesis, autophagy, mitophagy, angiogenesis, chemoresistance, cell proliferation and metabolism.^39^, ^40^, ^41^, ^42^ No studies have been conducted regarding the relationship between LAMP3 and NOD‐like receptor signalling. The relationship between LAMP3 and autophagy is not clear, and the study of it can be included in future research.

At last, we analysed the expression of LAMP3 and response to immunotherapy in tumours. LAMP3 levels correlated significantly with PD‐L1 in HNSC and BRCA. Our data confirm the potential ability of LAMP3 in the prediction of immunotherapy response and indicate that LAMP3 is a promising biomarker for cancer immunotherapy. However, because of our detailed results, further investigations of the predictive role of LAMP3 in cancer immunotherapy need to be performed clinically and mechanically in individual cancer types.

However, this study still has several limitations that need to be improved in the future. Firstly, we only validated the correlation between expression of LAMP3 and prognostic value in multiple analyses based on different databases, no clinical cohorts were available for further validation, which is urgently warranted in future experiments. Secondly, although LAMP3 was the crucial regulator in lysosomes and immune infiltration, other lysosome‐related genes may be related to tumorigenesis, which is essential to be researched in future analyses. Thirdly, some of our results are contradictory because of the heterogeneity of data across multiple databases.

## 5 CONCLUSION

In conclusion, our study systematically analysed LAMP3 expression and its relevant signalling pathways, genetic alteration and relation to immune infiltration and immunomodulation in pan‐cancer. As a result, we concluded that LAMP3 predicts the prognoses of cancer patients and immune cell infiltration across cancers in the future.

## AUTHOR CONTRIBUTIONS

**Xuefei Feng:** Writing – original draft (equal). **Lvye Gao:** Writing – review and editing (equal). **Xinyuan Shen:** Writing – review and editing (equal). **Mingtai Li:** Software (equal). **Xiaohui Wang:** Validation (equal). **Yanlong Hao:** Validation (equal). **Jinyan Chen:** Supervision (equal). **Yuanfang Zhai:** Supervision (equal). **Binbin Zou:** Software (equal). **Shangman Yao:** Supervision (equal). **Yanlin Guo:** Project administration (equal). **Ling Zhang:** Project administration (equal).

## FUNDING INFORMATION

This work was supported by the Science and Technology Innovation Team of Shanxi Province (202204051001024 to LZ), the Science and Technology Achievements Transformation Project of Shanxi Province (202204021301062 to LZ), Shanxi Scholarship Council of China (2021‐082 to LZ), Shanxi Province Science Foundation for Youths (20210302124185 to XFF), Scientific and Technological Innovation Programs of Higher Education Institutions in Shanxi (2021L208 to XFF).

## CONFLICT OF INTEREST STATEMENT

The authors confirm that there are no conflicts of interest.

## Supporting information


Figure S1.

Figure S2.

Figure S3.

Figure S4.

Figure S5.

Figure S6.

Figure S7.

Figure S8.

Figure S9.

Table S1.
Table S2.
Table S3.
Table S4.

## Data Availability

The datasets presented in this study can be found in online repositories. The names of the repository/repositories and accession number(s) can be found in the article/Supplementary Material.
