# Supplementary figures and images for: A pan‐cancer analysis of prognostic significance and immunological role of lysosomal‐associated membrane protein 3
Source: J Cell Mol Med. 2023 Dec 26;28(3):e18088. doi: 10.1111/jcmm.18088 (PMC10844704; doi:10.1111/jcmm.18088)

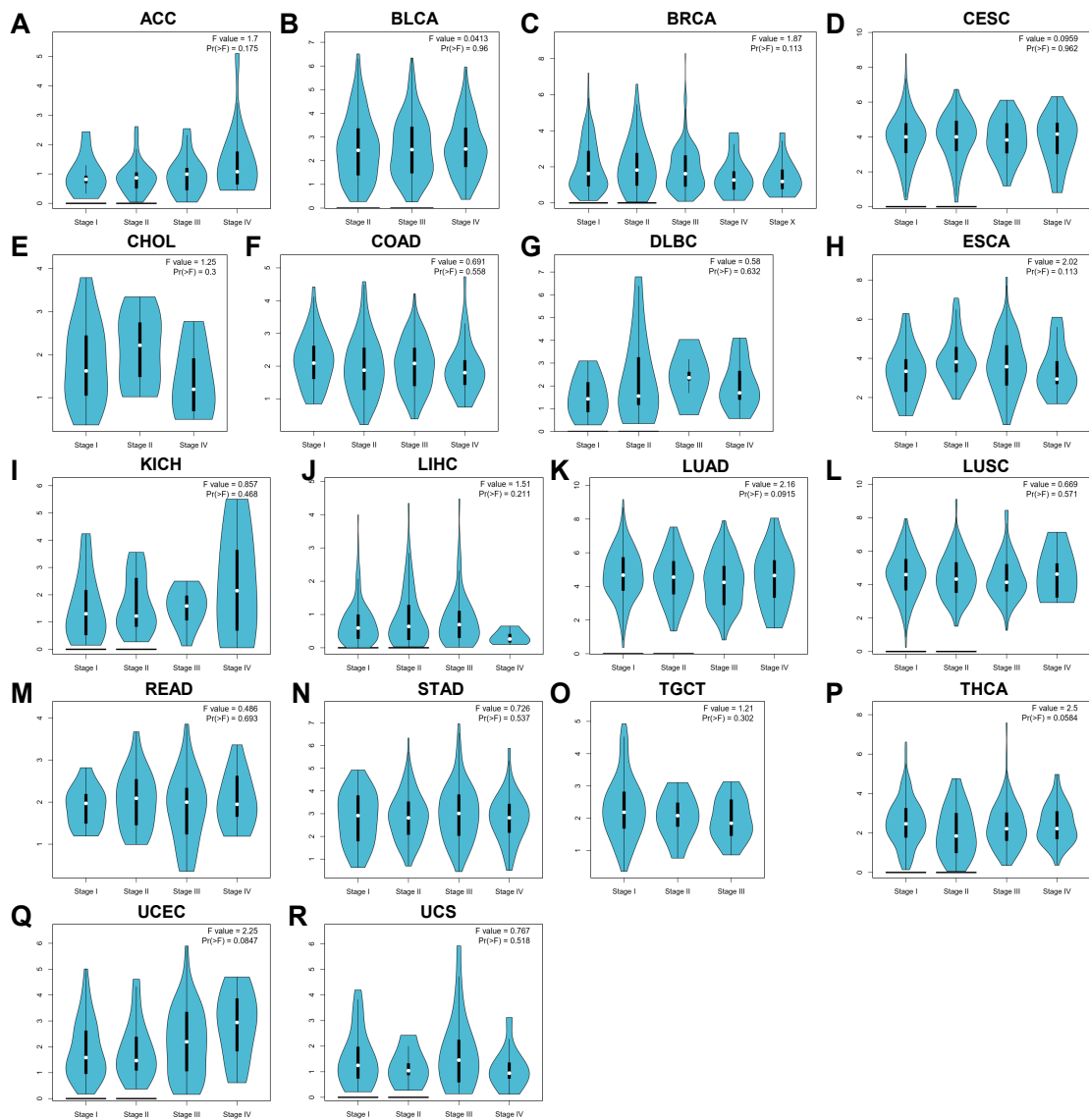

Supplement: Supplementary file 2 — Figure S2. [file JCMM-28-e18088-s009.pdf]

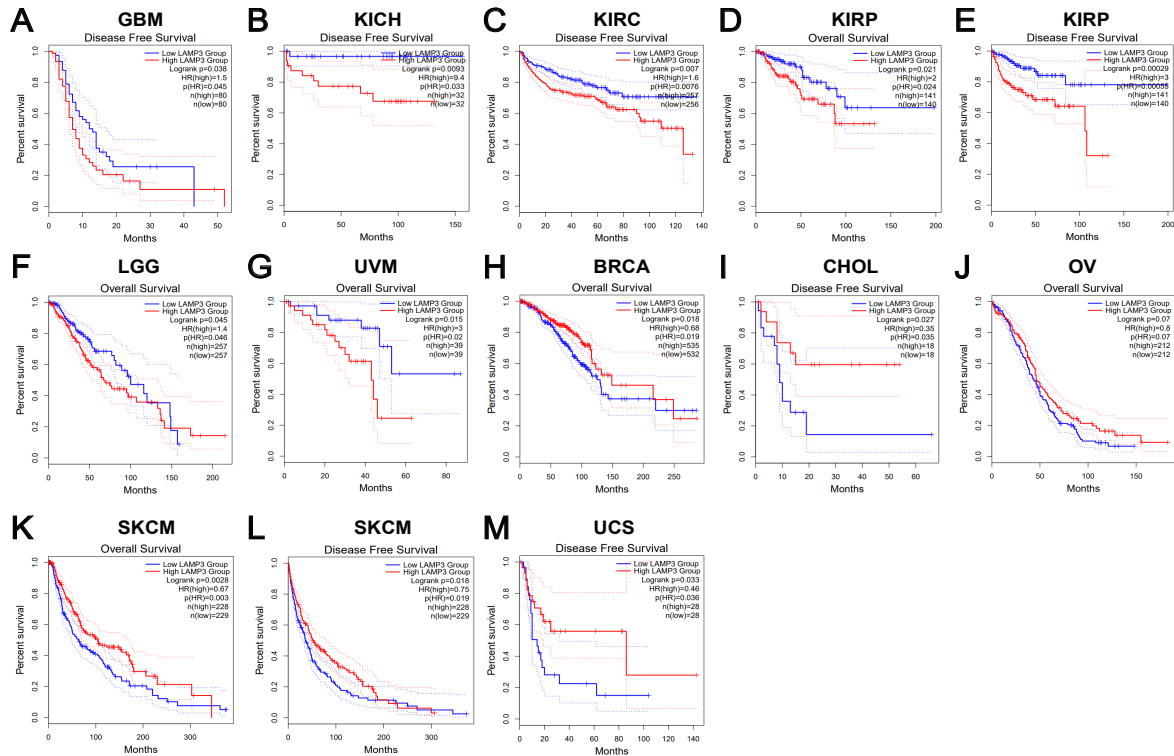

Supplement: Supplementary file 4 — Figure S4. [file JCMM-28-e18088-s005.pdf]

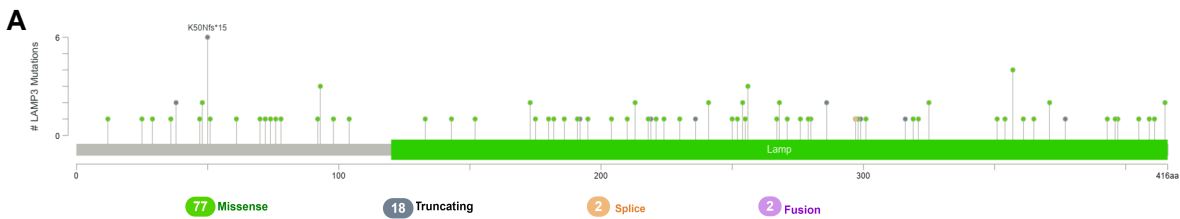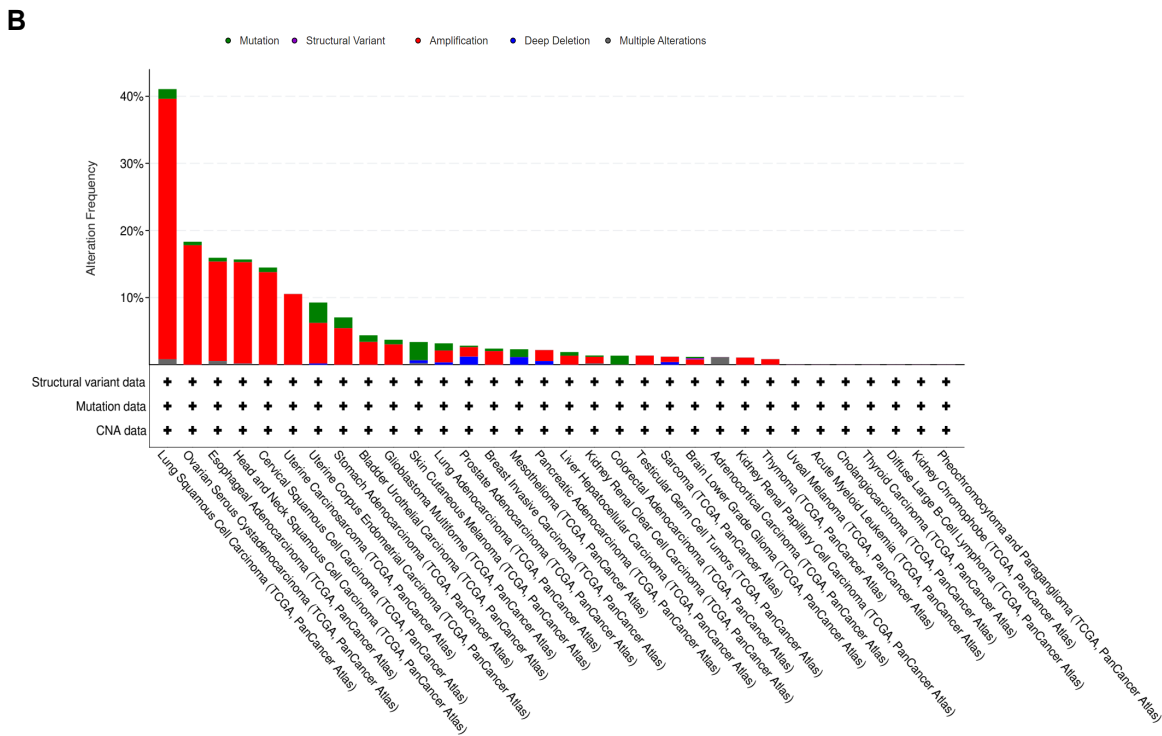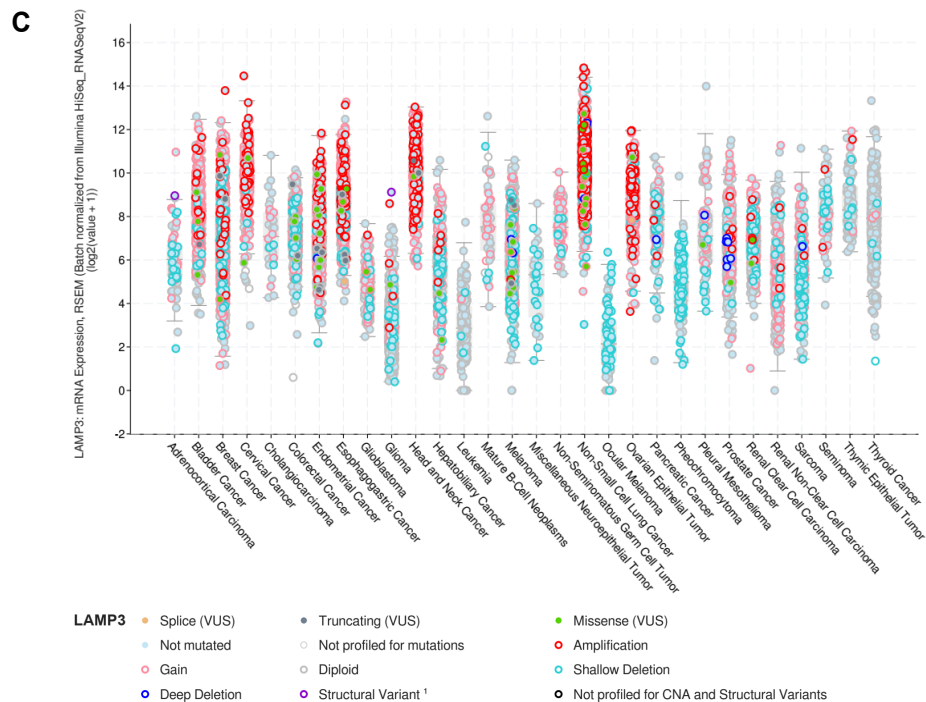

Supplement: Supplementary file 5 — Figure S5. [file JCMM-28-e18088-s003.pdf]

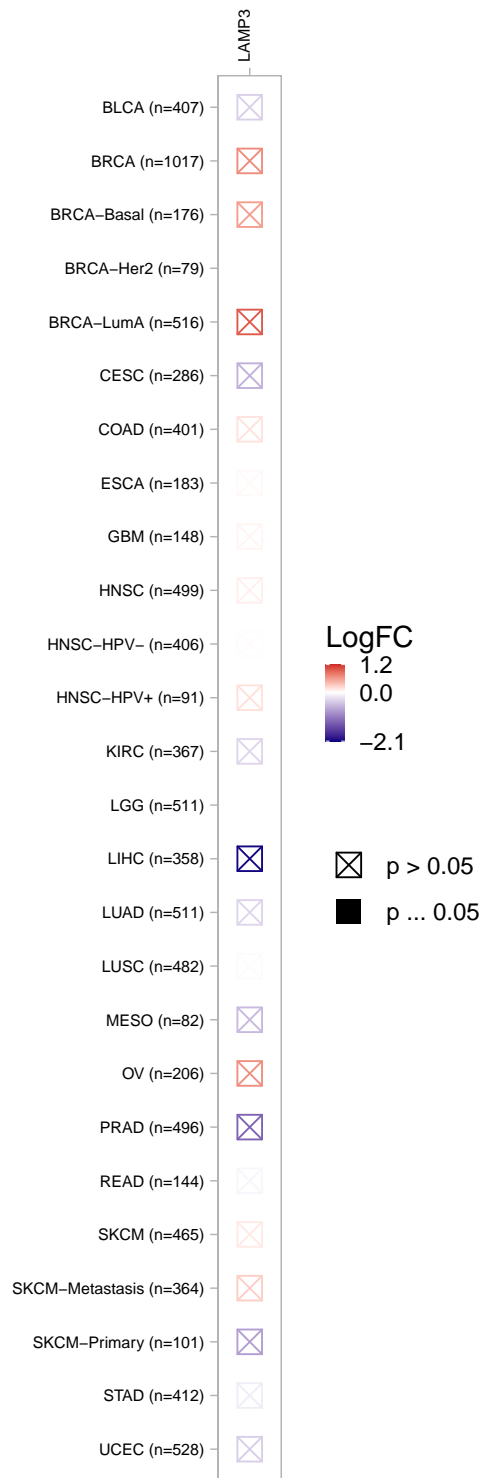

Supplement: Supplementary file 6 — Figure S6. [file JCMM-28-e18088-s008.pdf]

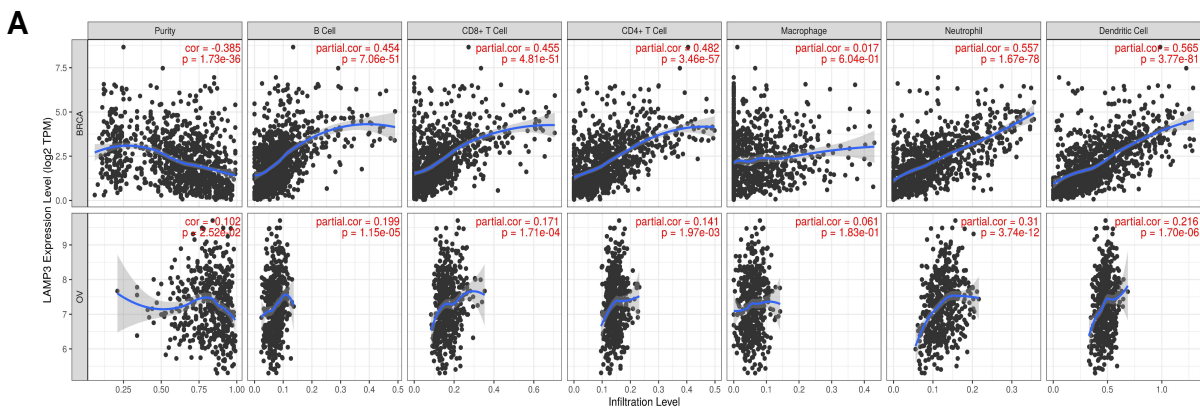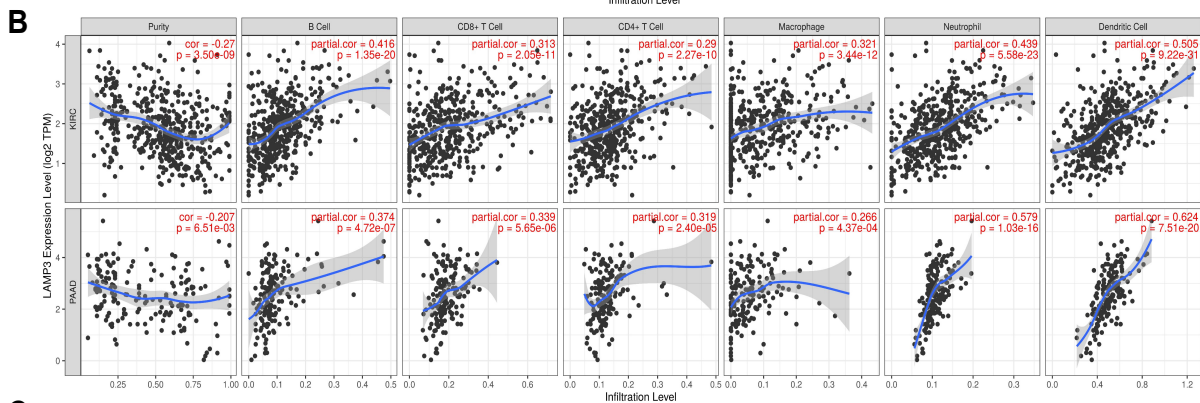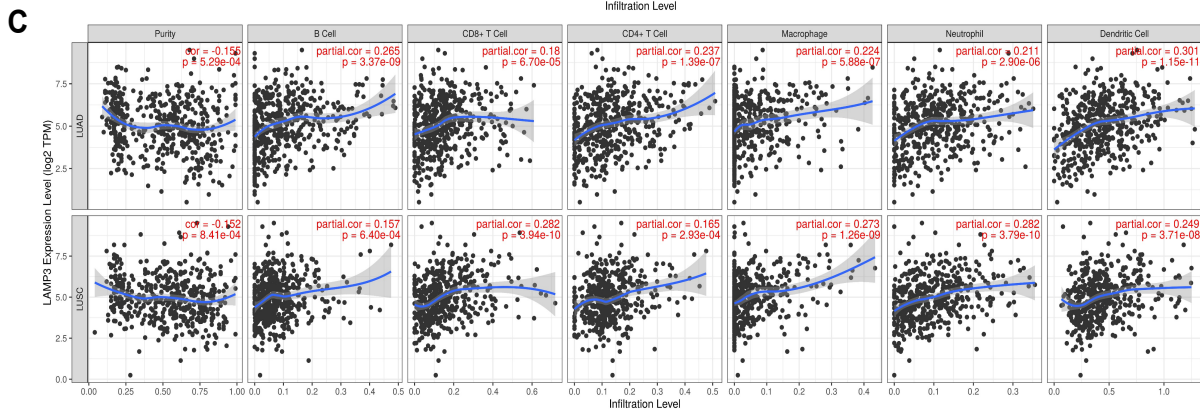

Supplement: Supplementary file 7 — Figure S7. [file JCMM-28-e18088-s006.pdf]

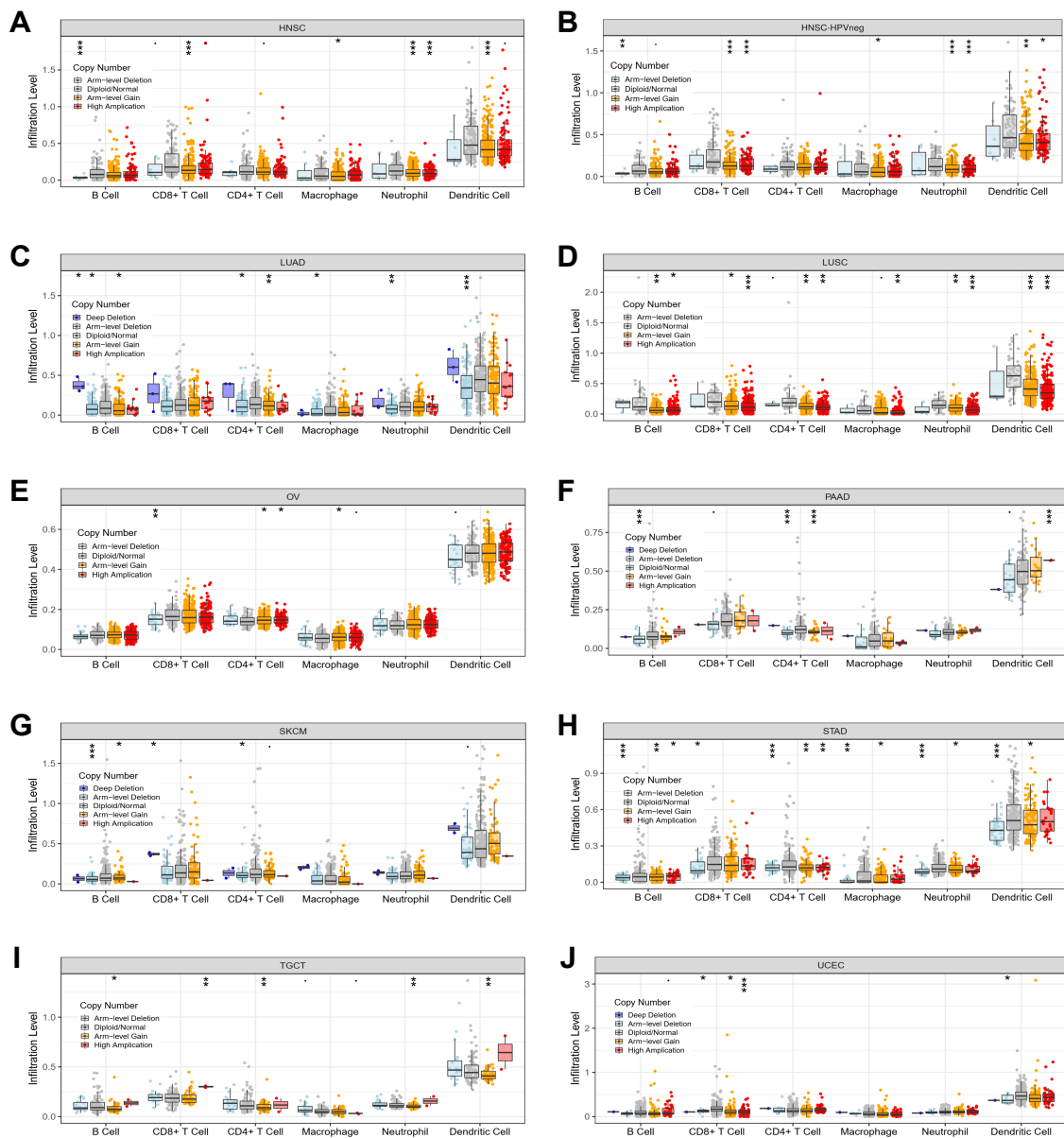

Supplement: Supplementary file 8 — Figure S8. [file JCMM-28-e18088-s001.pdf]

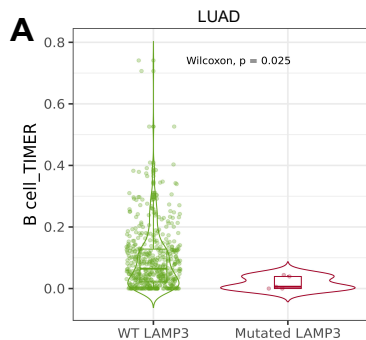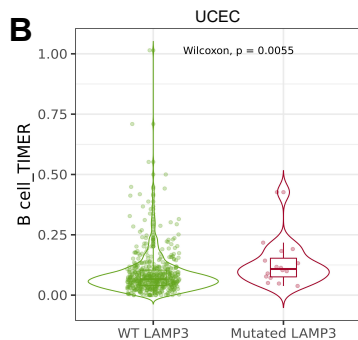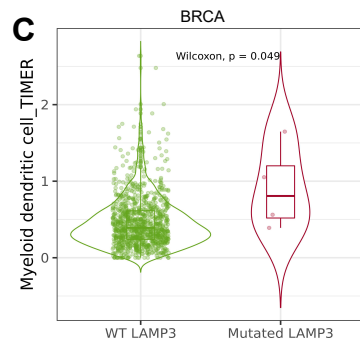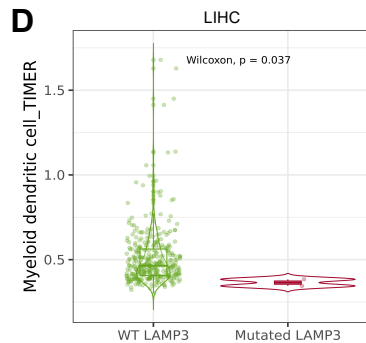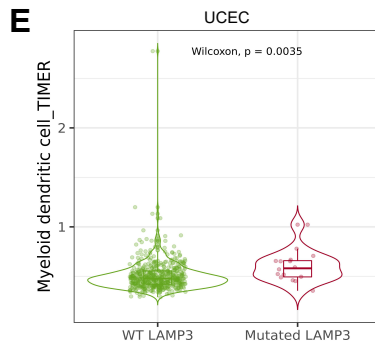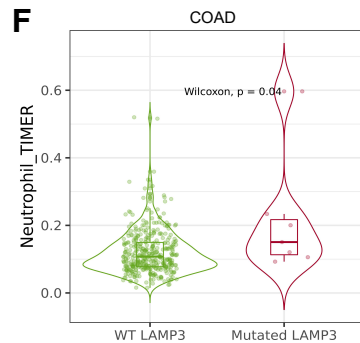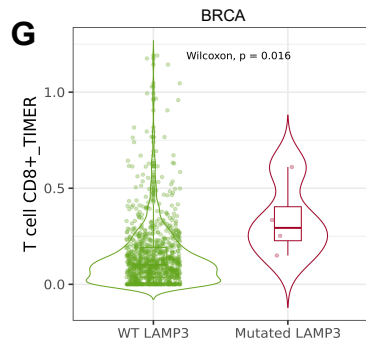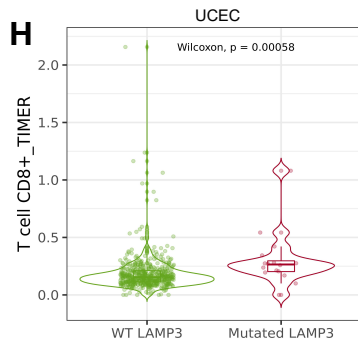

Supplement: Supplementary file 9 — Figure S9. [file JCMM-28-e18088-s007.pdf]
