# Supplementary material for: A pan‐cancer analysis of prognostic significance and immunological role of lysosomal‐associated membrane protein 3
Source: J Cell Mol Med. 2023 Dec 26;28(3):e18088. doi: 10.1111/jcmm.18088 (PMC10844704; doi:10.1111/jcmm.18088)
Supplement: Supplementary file 3 — Figure S3. [file JCMM-28-e18088-s002.pdf]

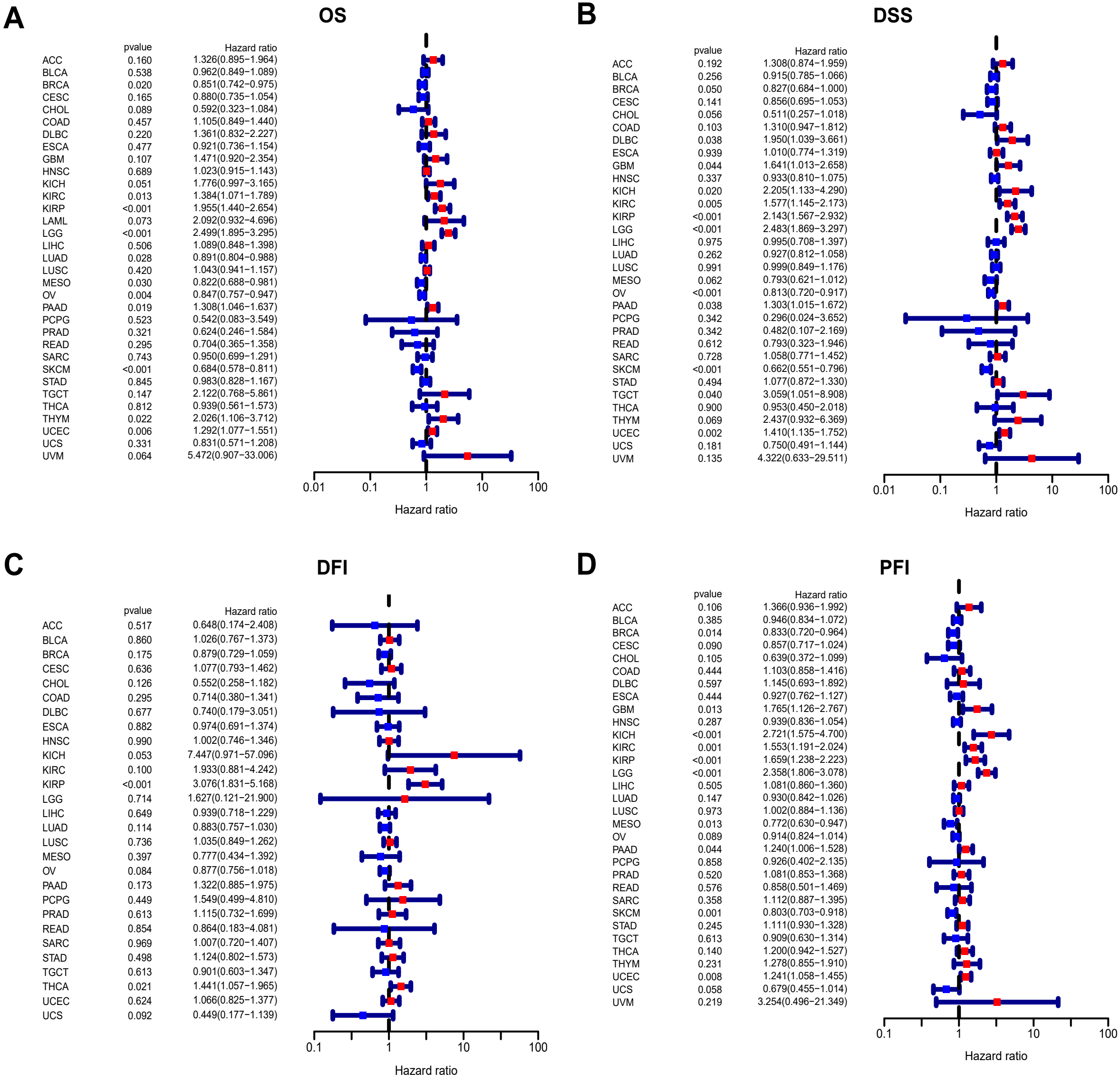

A
OS
pvalue
Hazard ratio
ACC 0.160 1.326(0.895−1.964)
BLCA 0.538 0.962(0.849−1.089)
BRCA 0.020 0.851(0.742−0.975)
CESC 0.165 0.880(0.735−1.054)
CHOL 0.089 0.592(0.323−1.084)
COAD 0.457 1.105(0.849−1.440)
DLBC 0.220 1.361(0.832−2.227)
ESCA 0.477 0.921(0.736−1.154)
GBM 0.107 1.471(0.920−2.354)
HNSC 0.689 1.023(0.915−1.143)
KICH 0.051 1.776(0.997−3.165)
KIRC 0.013 1.384(1.071−1.789)
KIRP <0.001 1.955(1.440−2.654)
LAML 0.073 2.092(0.932−4.696)
LGG <0.001 2.499(1.895−3.295)
LIHC 0.506 1.089(0.848−1.398)
LUAD 0.028 0.891(0.804−0.988)
LUSC 0.420 1.043(0.941−1.157)
MESO 0.030 0.822(0.688−0.981)
OV 0.004 0.847(0.757−0.947)
PAAD 0.019 1.308(1.046−1.637)
PCPG 0.523 0.542(0.083−3.549)
PRAD 0.321 0.624(0.246−1.584)
READ 0.295 0.704(0.365−1.358)
SARC 0.743 0.950(0.699−1.291)
SKCM <0.001 0.684(0.578−0.811)
STAD 0.845 0.983(0.828−1.167)
TGCT 0.147 2.122(0.768−5.861)
THCA 0.812 0.939(0.561−1.573)
THYM 0.022 2.026(1.106−3.712)
UCEC 0.006 1.292(1.077−1.551)
UCS 0.331 0.831(0.571−1.208)
UVM 0.064 5.472(0.907−33.006)
0.01 0.1 1 10 100
Hazard ratio
B
DSS
pvalue
Hazard ratio
ACC 0.192 1.308(0.874−1.959)
BLCA 0.256 0.915(0.785−1.066)
BRCA 0.050 0.827(0.684−1.000)
CESC 0.141 0.856(0.695−1.053)
CHOL 0.056 0.511(0.257−1.018)
COAD 0.103 1.310(0.947−1.812)
DLBC 0.038 1.950(1.039−3.661)
ESCA 0.939 1.010(0.774−1.319)
GBM 0.044 1.641(1.013−2.658)
HNSC 0.337 0.933(0.810−1.075)
KICH 0.020 2.205(1.133−4.290)
KIRC 0.005 1.577(1.145−2.173)
KIRP <0.001 2.143(1.567−2.932)
LGG <0.001 2.483(1.869−3.297)
LIHC 0.975 0.995(0.708−1.397)
LUAD 0.262 0.927(0.812−1.058)
LUSC 0.991 0.999(0.849−1.176)
MESO 0.062 0.793(0.621−1.012)
OV <0.001 0.813(0.720−0.917)
PAAD 0.038 1.303(1.015−1.672)
PCPG 0.342 0.296(0.024−3.652)
PRAD 0.342 0.482(0.107−2.169)
READ 0.612 0.793(0.323−1.946)
SARC 0.728 1.058(0.771−1.452)
SKCM <0.001 0.662(0.551−0.796)
STAD 0.494 1.077(0.872−1.330)
TGCT 0.040 3.059(1.051−8.908)
THCA 0.900 0.953(0.450−2.018)
THYM 0.069 2.437(0.932−6.369)
UCEC 0.002 1.410(1.135−1.752)
UCS 0.181 0.750(0.491−1.144)
UVM 0.135 4.322(0.633−29.511)
0.01 0.1 1 10 100
Hazard ratio
C
DFI
pvalue
Hazard ratio
ACC 0.517 0.648(0.174−2.408)
BLCA 0.860 1.026(0.767−1.373)
BRCA 0.175 0.879(0.729−1.059)
CESC 0.636 1.077(0.793−1.462)
CHOL 0.126 0.552(0.258−1.182)
COAD 0.295 0.714(0.380−1.341)
DLBC 0.677 0.740(0.179−3.051)
ESCA 0.882 0.974(0.691−1.374)
HNSC 0.990 1.002(0.746−1.346)
KICH 0.053 7.447(0.971−57.096)
KIRC 0.100 1.933(0.881−4.242)
KIRP <0.001 3.076(1.831−5.168)
LGG 0.714 1.627(0.121−21.900)
LIHC 0.649 0.939(0.718−1.229)
LUAD 0.114 0.883(0.757−1.030)
LUSC 0.736 1.035(0.849−1.262)
MESO 0.397 0.777(0.434−1.392)
OV 0.084 0.877(0.756−1.018)
PAAD 0.173 1.322(0.885−1.975)
PCPG 0.449 1.549(0.499−4.810)
PRAD 0.613 1.115(0.732−1.699)
READ 0.854 0.864(0.183−4.081)
SARC 0.969 1.007(0.720−1.407)
STAD 0.498 1.124(0.802−1.573)
TGCT 0.613 0.901(0.603−1.347)
THCA 0.021 1.441(1.057−1.965)
UCEC 0.624 1.066(0.825−1.377)
UCS 0.092 0.449(0.177−1.139)
0.1 1 10 100
Hazard ratio
D
PFI
pvalue
Hazard ratio
ACC 0.106 1.366(0.936−1.992)
BLCA 0.385 0.946(0.834−1.072)
BRCA 0.014 0.833(0.720−0.964)
CESC 0.090 0.857(0.717−1.024)
CHOL 0.105 0.639(0.372−1.099)
COAD 0.444 1.103(0.858−1.416)
DLBC 0.597 1.145(0.693−1.892)
ESCA 0.444 0.927(0.762−1.127)
GBM 0.013 1.765(1.126−2.767)
HNSC 0.287 0.939(0.836−1.054)
KICH <0.001 2.721(1.575−4.700)
KIRC 0.001 1.553(1.191−2.024)
KIRP <0.001 1.659(1.238−2.223)
LGG <0.001 2.358(1.806−3.078)
LIHC 0.505 1.081(0.860−1.360)
LUAD 0.147 0.930(0.842−1.026)
LUSC 0.973 1.002(0.884−1.136)
MESO 0.013 0.772(0.630−0.947)
OV 0.089 0.914(0.824−1.014)
PAAD 0.044 1.240(1.006−1.528)
PCPG 0.858 0.926(0.402−2.135)
PRAD 0.520 1.081(0.853−1.368)
READ 0.576 0.858(0.501−1.469)
SARC 0.358 1.112(0.887−1.395)
SKCM 0.001 0.803(0.703−0.918)
STAD 0.245 1.111(0.930−1.328)
TGCT 0.613 0.909(0.630−1.314)
THCA 0.140 1.200(0.942−1.527)
THYM 0.231 1.278(0.855−1.910)
UCEC 0.008 1.241(1.058−1.455)
UCS 0.058 0.679(0.455−1.014)
UVM 0.219 3.254(0.496−21.349)
0.1 1 10 100
Hazard ratio
