# Supplementary material for: A pan‐cancer analysis of prognostic significance and immunological role of lysosomal‐associated membrane protein 3
Source: J Cell Mol Med. 2023 Dec 26;28(3):e18088. doi: 10.1111/jcmm.18088 (PMC10844704; doi:10.1111/jcmm.18088)
Supplement: Supplementary file 10 — Table S1. Table S2. Table S3. Table S4. [file JCMM-28-e18088-s004.pdf]

Supplementary Table 1. Abbreviations and full names of 33 tumors

Abbreviation	Full name
ACC	Adrenocortical carcinoma
BLCA	Bladder Urothelial Carcinoma
BRCA	Breast invasive carcinoma
CESC	Cervical squamous cell carcinoma and endocervical adenocarcinoma
CHOL	Cholangiocarcinoma
COAD	Colon adenocarcinoma
DLBC	Lymphoid Neoplasm Diffuse Large B-cell Lymphoma
ESCA	Esophageal carcinoma
GBM	Glioblastoma multiforme
HNSC	Head and Neck squamous cell carcinoma
KICH	Kidney Chromophobe
KIRC	Kidney renal clear cell carcinoma
KIRP	Kidncy renal papillary cell carcinoma
LAML	Acute Mycloid Loukemia
LGG	Brain Lower Grade Glioma
LIHC	Liver hcpatocellular carcinoma
LUAD	Lung adenocarcinoma
LUSC	Lung squamous cell carcinoma
MESO	Mesothelioma
OV	Ovarian carcinoma
PAAD	Pancreatic adenocarcinoma
PCPG	Phcochromocytoma and Paraganglioma
PRAD	Prostate adenocarcinoma
READ	Rectum adenocarcinoma
SARC	Sarcoma
SKCM	Skin Cutancous Melanoma
STAD	Stomach adenocarcinoma
TGCT	Testicular Germ Cell Tumors
THCA	Thyroid Carcinoma
THYM	Thymoma
UCEC	Uterine Corpus Endometrial Carcinoma

UCS	Uterine Carcinosarcoma
UVM	Uvcal Melanoma

Supplementary Table 2. Survival analyses of LAMP3 expression in pan-cancer (in Kaplan Meier)

Cancer type	Survival outcome	HR	95%CI	*P*-value
Bladder Carcinoma (BLCA)	OS	0.78	0.57-1.07	0.12
	RFS	0.72	0.35-1.46	0.36
Breast cancer (BRCA)	OS	0.59	0.43-0.83	0.002
	RFS	0.56	0.36-0.88	0.011
Cervical squamous cell carcinoma (CESC)	OS	0.6	0.36-0.99	0.044
	RFS	1.98	0.68-5.74	0.2
Esophageal Adenocarcinoma (ESCA)	OS	0.56	0.29-1.07	0.076
	RFS	3.46	0.49-24.7	0.19
Esophageal Squamous Cell Carcinoma (ESCC)	OS	0.59	0.26-1.34	0.2
	RFS	0.38	0.12-1.18	0.082
Head-neck squamous cell carcinoma (HNSC)	OS	0.84	0.64-1.1	0.21
	RFS	0.6	0.28-1.28	0.18
Kidney renal clear cell carcinoma (KIRC)	OS	1.71	1.16-2.52	0.0065
	RFS	340774563	0-lnf	0.0074
Kidney renal papillary cell carcinoma (KIRP)	OS	4.29	2.28-8.04	8.40E-07
	RFS	4.04	1.78-9.2	0.00031
Liver hepatocellular carcinoma (LIHC)	OS	1.35	0.94-1.95	0.1
	RFS	0.78	0.55-1.11	0.17
Lung adenocarcinoma (LUAD)	OS	0.67	0.5-0.9	0.0068
	RFS	0.6	0.39-0.91	0.015
Lung squamous cell carcinoma (LUSC)	OS	1.33	1.01-1.74	0.042
	RFS	0.58	0.35-0.96	0.032
Ovarian cancer (OV)	OS	0.62	0.46-0.83	0.0014
	RFS	0.67	0.45-0.98	0.04
Pancreatic ductal adenocarcinoma (PAAD)	OS	1.94	1.16-3.25	0.011
	RFS	2.67	1-7.09	0.042
Pheochromocytoma and Paraganglioma (PCPG)	OS	2.95	0.34-25.3	0.3
	RFS	8.91	0.92-86.0	0.022

Rectum adenocarcinoma (READ)	OS	0.45	0.21-0.99	0.042
	RFS	0.17	0.03-1.03	0.029
Sarcoma (SARC)	OS	0.75	0.5-1.11	0.15
	RFS	1.25	0.77-2.03	0.37
Stomach adenocarcinoma (STAD)	OS	0.85	0.58-1.25	0.4
	RFS	1.76	0.92-3.37	0.085
Testicular Germ Cell Tumor (TGCT)	OS	7.45	0.77-71.4	0.041
	RFS	0.64	0.3-1.36	0.24
Thymoma (THYM)	OS	6.86	0.84-55.9	0.039
Thyroid carcinoma (THCA)	OS	0.43	0.16-1.15	0.084
	RFS	2.68	1.24-5.8	0.0091
Uterine corpus endometrial carcinoma (UCEC)	OS	1.92	1.25-2.94	0.0022
	RFS	1.67	0.99-2.82	0.05

Supplementary Table 3. Survival analyses of LAMP3 expression in pan-cancer (in GEPIA)

Cancer Type	Survival outcome	N	HR	*P*-value
Adrenocortical carcinoma (ACC)	OS	76	1.4	0.4
	DFS	76	1.7	0.13
Bladder Urothelial Carcinoma (BLCA)	OS	402	1	0.91
	DFS	402	1	0.81
Breast invasive carcinoma (BRCA)	OS	1067	0.68	0.018
	DFS	1067	0.7	0.06
Cervical squamous cell carcinoma and endocervical adenocarcinoma (CESC)	OS	292	0.83	0.45
	DFS	292	0.73	0.28
Cholangiocarcinoma (CHOL)	OS	36	0.38	0.061
	DFS	36	0.35	0.027
Colon adenocarcinoma (COAD)	OS	270	1.2	0.56
	DFS	270	1.2	0.36
Lymphoid Neoplasm Diffuse Large B-cell Lymphoma (DLBC)	OS	46	1.6	0.5
	DFS	46	0.97	0.95
Esophageal carcinoma (ESCA)	OS	182	0.8	0.34
	DFS	182	0.9	0.65
Glioblastoma multiforme (GBM)	OS	160	1.3	0.22
	DFS	160	1.5	0.038
Head and Neck squamous cell carcinoma (HNSC)	OS	518	0.99	0.96
	DFS	518	0.99	0.96
Kidney Chromophobe (KICH)	OS	64	3.3	0.11

	DFS	64	9.4	0.0093
Kidney renal clear cell carcinoma (KIRC)	OS	513	1.1	0.48
	DFS	513	1.6	0.007
Kidncy renal papillary cell carcinoma (KIRP)	OS	281	2	0.021
	DFS	281	3	0.00029
Acute Mycloid Loukemia (LAML)	OS	106	1.3	0.37
	DFS	106	1	1
Brain Lower Grade Glioma (LGG)	OS	514	1.4	0.045
	DFS	514	1.2	0.19
Liver hcpatocellular carcinoma (LIHC)	OS	364	1.1	0.44
	DFS	364	1.1	0.47
Lung adenocarcinoma (LUAD)	OS	478	0.83	0.21
	DFS	478	0.83	0.23
Lung squamous cell carcinoma (LUSC)	OS	281	1.2	0.28
	DFS	281	1	0.94
Mesothelioma (MESO)	OS	82	0.74	0.21
	DFS	82	0.76	0.34
Ovarian serous cystadenocarcinoma (OV)	OS	424	0.8	0.07
	DFS	424	0.9	0.42
Pancreatic adenocarcinoma (PAAD)	OS	178	1.2	0.43
	DFS	178	1.2	0.41
Phcochromocytoma and Paraganglioma (PCPG)	OS	180	0.89	0.89
	DFS	180	0.96	0.93
Prostate adenocarcinoma (PRAD)	OS	490	0.86	0.81
	DFS	490	0.89	0.59
Rectum adenocarcinoma (READ)	OS	92	0.98	0.96
	DFS	92	0.79	0.62
Sarcoma (SARC)	OS	262	0.95	0.81
	DFS	262	1.1	0.53
Skin Cutancous Melanoma (SKCM)	OS	457	0.67	0.0028
	DFS	457	0.75	0.018
Stomach adenocarcinoma (STAD)	OS	384	1	0.85
	DFS	384	1.1	0.57
Testicular Germ Cell Tumors (TGCT)	OS	135	4.1	0.2
	DFS	135	0.75	0.42
Thyroid carcinoma (THCA)	OS	510	1.1	0.82
	DFS	510	1.3	0.83
Thymoma (THYM)	OS	118	1.4	0.67
	DFS	118	1.1	0.88
Uterine Corpus Endometrial Carcinoma (UCEC)	OS	172	1.6	0.19
	DFS	172	1.4	0.33
Uterine Carcinosarcoma (UCS)	OS	56	0.68	0.62
	DFS	56	0.46	0.033
Uvcal Melanoma (UVM)	OS	78	3	0.015

	DFS	78	1.8	0.22

Supplementary Table 4. The correlation between LAMP3 expression and immune infiltration level of B cell, CD8+ T cell, CD4+ T cell, macrophage, neutrophil and DCs in Pan-cancer in TIMER.

cancer	variable	partial.cor	*P*
ACC	Purity	-0.125810387	0.285481057
ACC	B Cell	0.491659216	1.00E-05
ACC	CD8+ T Cell	0.114052638	0.336659324
ACC	CD4+ T Cell	0.084025692	0.479705627
ACC	Macrophage	0.172587397	0.144257727
ACC	Neutrophil	0.327953177	0.004619138
ACC	Dendritic Cell	0.384186331	0.000791918
BLCA	Purity	-0.370144632	2.00E-13
BLCA	B Cell	0.124220151	0.017897765
BLCA	CD8+ T Cell	0.221182253	1.95E-05
BLCA	CD4+ T Cell	0.268994107	1.81E-07
BLCA	Macrophage	-0.020812929	0.691876735
BLCA	Neutrophil	0.481928267	1.65E-22
BLCA	Dendritic Cell	0.393833622	5.43E-15
BRCA	Purity	-0.384916441	1.73E-36
BRCA	B Cell	0.45412964	7.06E-51
BRCA	CD8+ T Cell	0.455020572	4.81E-51
BRCA	CD4+ T Cell	0.482275172	3.46E-57
BRCA	Macrophage	0.016545834	0.604364678
BRCA	Neutrophil	0.556706941	1.67E-78
BRCA	Dendritic Cell	0.564545642	3.77E-81
BRCA-Basal	Purity	-0.465912836	2.63E-08
BRCA-Basal	B Cell	0.425597221	8.32E-07
BRCA-Basal	CD8+ T Cell	0.334887057	0.000153043
BRCA-Basal	CD4+ T Cell	0.367269692	3.17E-05
BRCA-Basal	Macrophage	0.035153772	0.694786918
BRCA-Basal	Neutrophil	0.504838911	1.87E-08
BRCA-Basal	Dendritic Cell	0.473213431	1.06E-07
BRCA-Her2	Purity	-0.504977315	4.53E-05
BRCA-Her2	B Cell	0.376493835	0.003581094
BRCA-Her2	CD8+ T Cell	0.458592476	0.000333728
BRCA-Her2	CD4+ T Cell	0.51890876	3.00E-05
BRCA-Her2	Macrophage	-0.072805883	0.587044053
BRCA-Her2	Neutrophil	0.593714715	9.00E-07
BRCA-Her2	Dendritic Cell	0.449440243	0.000511158
BRCA-Luminal	Purity	-0.388674731	3.92E-21
BRCA-Luminal	B Cell	0.410350281	2.18E-23

BRCA-Luminal	CD8+ T Cell	0.593290133	2.29E-52
BRCA-Luminal	CD4+ T Cell	0.542471336	2.44E-42
BRCA-Luminal	Macrophage	0.142187145	0.000901848
BRCA-Luminal	Neutrophil	0.560340748	1.79E-45
BRCA-Luminal	Dendritic Cell	0.592252022	5.89E-52
CESC	Purity	-0.264740877	7.67E-06
CESC	B Cell	0.065526349	0.277119638
CESC	CD8+ T Cell	0.222150693	0.000209843
CESC	CD4+ T Cell	0.357913688	8.55E-10
CESC	Macrophage	-0.199286205	0.000852067
CESC	Neutrophil	0.400784691	4.12E-12
CESC	Dendritic Cell	0.394523658	1.03E-11
CHOL	Purity	-0.276004129	0.103226795
CHOL	B Cell	0.385031505	0.022371188
CHOL	CD8+ T Cell	0.398079648	0.017866983
CHOL	CD4+ T Cell	0.570790234	0.000342847
CHOL	Macrophage	0.456559386	0.005835859
CHOL	Neutrophil	0.281823114	0.100968814
CHOL	Dendritic Cell	0.3675818	0.029828557
COAD	Purity	-0.235980986	1.48E-06
COAD	B Cell	0.252617646	2.67E-07
COAD	CD8+ T Cell	0.43681546	2.40E-20
COAD	CD4+ T Cell	0.412659427	5.83E-18
COAD	Macrophage	0.408150458	1.19E-17
COAD	Neutrophil	0.601063338	9.55E-41
COAD	Dendritic Cell	0.596269571	4.60E-40
DLBC	Purity	-0.738864395	2.31E-08
DLBC	B Cell	-0.04995828	0.843936652
DLBC	CD8+ T Cell	-0.168951956	0.464098585
DLBC	CD4+ T Cell	0.280613531	0.217899267
DLBC	Macrophage	0.052493631	0.821215959
DLBC	Neutrophil	0.410182355	0.064771967
DLBC	Dendritic Cell	0.190415037	0.408370632
ESCA	Purity	-0.151255382	0.042098618
ESCA	B Cell	0.106383382	0.156375759
ESCA	CD8+ T Cell	-0.021919594	0.770234237
ESCA	CD4+ T Cell	0.062535255	0.405624862
ESCA	Macrophage	0.061964702	0.408607604
ESCA	Neutrophil	0.124809104	0.09504646
ESCA	Dendritic Cell	0.215096726	0.003734712
GBM	Purity	-0.226576853	2.80E-06
GBM	B Cell	-0.022656447	0.644164265
GBM	CD8+ T Cell	-0.247030083	3.14E-07
GBM	CD4+ T Cell	-0.098984209	0.043109618

GBM	Macrophage	-0.065614967	0.180594371
GBM	Neutrophil	-0.047029543	0.337471721
GBM	Dendritic Cell	0.214733513	9.47E-06
HNSC	Purity	-0.136555031	0.002377097
HNSC	B Cell	0.125438025	0.006136994
HNSC	CD8+ T Cell	0.264877831	4.73E-09
HNSC	CD4+ T Cell	0.446978727	5.94E-25
HNSC	Macrophage	0.185171263	4.23E-05
HNSC	Neutrophil	0.604308955	5.13E-49
HNSC	Dendritic Cell	0.413401047	2.55E-21
HNSC-HPVpos	Purity	-0.105199887	0.323738445
HNSC-HPVpos	B Cell	0.011434295	0.920333547
HNSC-HPVpos	CD8+ T Cell	0.30792863	0.006094991
HNSC-HPVpos	CD4+ T Cell	0.336035054	0.00202532
HNSC-HPVpos	Macrophage	-0.012275058	0.910686495
HNSC-HPVpos	Neutrophil	0.594791431	1.94E-09
HNSC-HPVpos	Dendritic Cell	0.318579715	0.003332558
HNSC-HPVneg	Purity	-0.180024259	0.000290784
HNSC-HPVneg	B Cell	0.126067122	0.012265595
HNSC-HPVneg	CD8+ T Cell	0.228757466	4.62E-06
HNSC-HPVneg	CD4+ T Cell	0.465586617	1.21E-22
HNSC-HPVneg	Macrophage	0.222601948	8.17E-06
HNSC-HPVneg	Neutrophil	0.603349102	3.91E-40
HNSC-HPVneg	Dendritic Cell	0.422530139	1.40E-18
KICH	Purity	0.126221506	0.312551849
KICH	B Cell	0.348462696	0.004446301
KICH	CD8+ T Cell	0.070378884	0.577462794
KICH	CD4+ T Cell	0.051245851	0.685178199
KICH	Macrophage	-0.09817332	0.43655539
KICH	Neutrophil	0.192783441	0.123905836
KICH	Dendritic Cell	0.284979892	0.021395148
KIRC	Purity	-0.270387184	3.50E-09
KIRC	B Cell	0.415615433	1.35E-20
KIRC	CD8+ T Cell	0.313067769	2.05E-11
KIRC	CD4+ T Cell	0.290090698	2.27E-10
KIRC	Macrophage	0.320916458	3.44E-12
KIRC	Neutrophil	0.438824203	5.58E-23
KIRC	Dendritic Cell	0.504575625	9.22E-31
KIRP	Purity	-0.173630995	0.005076715
KIRP	B Cell	0.543556502	4.41E-21
KIRP	CD8+ T Cell	0.557133222	1.94E-22
KIRP	CD4+ T Cell	0.233083542	0.000158181
KIRP	Macrophage	0.142648578	0.024375722
KIRP	Neutrophil	0.358435547	3.08E-09

KIRP	Dendritic Cell	0.472356404	1.24E-15
LGG	Purity	-0.158488076	0.000498339
LGG	B Cell	0.362936642	2.50E-16
LGG	CD8+ T Cell	0.300845503	1.86E-11
LGG	CD4+ T Cell	0.499255748	2.26E-31
LGG	Macrophage	0.547011804	2.88E-38
LGG	Neutrophil	0.572863216	8.93E-43
LGG	Dendritic Cell	0.561542776	6.63E-41
LIHC	Purity	-0.188184303	0.000432759
LIHC	B Cell	0.559638174	9.61E-30
LIHC	CD8+ T Cell	0.398470928	1.84E-14
LIHC	CD4+ T Cell	0.495281545	1.08E-22
LIHC	Macrophage	0.458440089	4.01E-19
LIHC	Neutrophil	0.495594273	8.76E-23
LIHC	Dendritic Cell	0.528484472	7.44E-26
LUAD	Purity	-0.155361733	0.000529437
LUAD	B Cell	0.264620651	3.37E-09
LUAD	CD8+ T Cell	0.179637681	6.70E-05
LUAD	CD4+ T Cell	0.236614581	1.39E-07
LUAD	Macrophage	0.224490726	5.88E-07
LUAD	Neutrophil	0.21098873	2.90E-06
LUAD	Dendritic Cell	0.300845728	1.15E-11
LUSC	Purity	-0.152232236	0.000840647
LUSC	B Cell	0.156587982	0.000640108
LUSC	CD8+ T Cell	0.281966096	3.94E-10
LUSC	CD4+ T Cell	0.165460681	0.00029276
LUSC	Macrophage	0.273418194	1.26E-09
LUSC	Neutrophil	0.281935579	3.79E-10
LUSC	Dendritic Cell	0.24945525	3.71E-08
MESO	Purity	-0.296574557	0.005558513
MESO	B Cell	0.394465947	0.00020488
MESO	CD8+ T Cell	0.197955222	0.071072845
MESO	CD4+ T Cell	-0.013104508	0.90582186
MESO	Macrophage	-0.228131296	0.036875826
MESO	Neutrophil	0.425773694	5.40E-05
MESO	Dendritic Cell	0.300980396	0.005402194
OV	Purity	-0.101719981	0.025230155
OV	B Cell	0.198760262	1.15E-05
OV	CD8+ T Cell	0.17070007	0.000171472
OV	CD4+ T Cell	0.14092553	0.001968576
OV	Macrophage	0.06085629	0.183171847
OV	Neutrophil	0.310028336	3.74E-12
OV	Dendritic Cell	0.216439185	1.70E-06
PAAD	Purity	-0.206744405	0.006506136

PAAD	B Cell	0.373888251	4.72E-07
PAAD	CD8+ T Cell	0.339260536	5.65E-06
PAAD	CD4+ T Cell	0.318769963	2.40E-05
PAAD	Macrophage	0.266005055	0.000437233
PAAD	Neutrophil	0.579370554	1.03E-16
PAAD	Dendritic Cell	0.624139885	7.51E-20
PCPG	Purity	-0.16706491	0.030427756
PCPG	B Cell	0.09049381	0.244812406
PCPG	CD8+ T Cell	-0.060177777	0.439801932
PCPG	CD4+ T Cell	0.297570195	9.41E-05
PCPG	Macrophage	0.277126019	0.000300816
PCPG	Neutrophil	0.205115046	0.007835076
PCPG	Dendritic Cell	0.198752583	0.010026935
PRAD	Purity	-0.363179679	1.90E-14
PRAD	B Cell	0.539393557	1.78E-32
PRAD	CD8+ T Cell	0.488889034	2.22E-26
PRAD	CD4+ T Cell	0.498669512	3.18E-27
PRAD	Macrophage	0.542049015	3.82E-33
PRAD	Neutrophil	0.639716379	4.99E-49
PRAD	Dendritic Cell	0.579867275	1.18E-38
READ	Purity	-0.315076912	0.000149645
READ	B Cell	0.174813259	0.039562913
READ	CD8+ T Cell	0.305056073	0.0002607
READ	CD4+ T Cell	0.147606093	0.082909759
READ	Macrophage	0.055491743	0.516448075
READ	Neutrophil	0.260467339	0.002032751
READ	Dendritic Cell	0.374952107	5.42E-06
SARC	Purity	-0.341286324	4.25E-08
SARC	B Cell	0.50078284	1.41E-16
SARC	CD8+ T Cell	0.383662925	7.77E-10
SARC	CD4+ T Cell	0.297130797	2.93E-06
SARC	Macrophage	0.231429341	0.000346873
SARC	Neutrophil	0.387481738	4.32E-10
SARC	Dendritic Cell	0.504499948	5.76E-17
SKCM	Purity	-0.549803302	1.54E-37
SKCM	B Cell	0.295027309	1.90E-10
SKCM	CD8+ T Cell	0.500001629	4.40E-29
SKCM	CD4+ T Cell	0.265690043	1.21E-08
SKCM	Macrophage	0.179709599	0.000120215
SKCM	Neutrophil	0.525257699	2.03E-33
SKCM	Dendritic Cell	0.489344508	3.12E-28
SKCM-Primary	Purity	-0.534086701	6.25E-09
SKCM-Primary	B Cell	0.037335687	0.710877495
SKCM-Primary	CD8+ T Cell	0.484518125	2.84E-07

SKCM-Primary	CD4+ T Cell	0.283268326	0.004099325
SKCM-Primary	Macrophage	0.296058143	0.002647034
SKCM-Primary	Neutrophil	0.576917741	3.34E-10
SKCM-Primary	Dendritic Cell	0.251027676	0.011338893
SKCM-Metastasis	Purity	-0.558803207	1.52E-30
SKCM-Metastasis	B Cell	0.341234095	6.98E-11
SKCM-Metastasis	CD8+ T Cell	0.499319439	1.38E-22
SKCM-Metastasis	CD4+ T Cell	0.249911247	2.69E-06
SKCM-Metastasis	Macrophage	0.134520772	0.011644705
SKCM-Metastasis	Neutrophil	0.526188103	2.18E-26
SKCM-Metastasis	Dendritic Cell	0.543296568	8.30E-28
STAD	Purity	-0.151982013	0.002976185
STAD	B Cell	-0.140191651	0.006992756
STAD	CD8+ T Cell	0.408564074	2.56E-16
STAD	CD4+ T Cell	0.180000439	0.000539905
STAD	Macrophage	0.159470835	0.002092239
STAD	Neutrophil	0.565334811	1.02E-32
STAD	Dendritic Cell	0.489935014	8.52E-24
TGCT	Purity	-0.425857475	6.81E-08
TGCT	B Cell	0.366101646	5.11E-06
TGCT	CD8+ T Cell	0.045888456	0.581010564
TGCT	CD4+ T Cell	0.577918616	2.21E-14
TGCT	Macrophage	0.202832541	0.013743933
TGCT	Neutrophil	0.641058164	2.21E-18
TGCT	Dendritic Cell	0.548784404	7.43E-13
THCA	Purity	-0.116753184	0.009765086
THCA	B Cell	0.401674964	4.10E-20
THCA	CD8+ T Cell	0.091887327	0.042677215
THCA	CD4+ T Cell	0.415357656	8.93E-22
THCA	Macrophage	0.389505633	3.97E-19
THCA	Neutrophil	0.512474359	4.94E-34
THCA	Dendritic Cell	0.502030004	2.51E-32
THYM	Purity	0.143054132	0.125536004
THYM	B Cell	-0.180773568	0.054260485
THYM	CD8+ T Cell	-0.353443785	0.000114585
THYM	CD4+ T Cell	-0.429787008	2.51E-06
THYM	Macrophage	-0.489487352	3.26E-08
THYM	Neutrophil	0.184792499	0.049035122
THYM	Dendritic Cell	-0.396411002	1.26E-05
UCEC	Purity	-0.057883266	0.322617768
UCEC	B Cell	0.338342711	3.59E-09
UCEC	CD8+ T Cell	0.0485045	0.411369203
UCEC	CD4+ T Cell	0.378008493	2.56E-11
UCEC	Macrophage	0.090081441	0.124579151

UCEC	Neutrophil	0.517857932	1.67E-21
UCEC	Dendritic Cell	0.314359156	4.05E-08
UCS	Purity	-0.171234687	0.215702018
UCS	B Cell	0.331393389	0.015351387
UCS	CD8+ T Cell	0.471597745	0.000364393
UCS	CD4+ T Cell	0.105860651	0.450594809
UCS	Macrophage	0.147188768	0.292918586
UCS	Neutrophil	0.616646892	8.81E-07
UCS	Dendritic Cell	0.489017729	0.000202729
UVM	Purity	0.136002886	0.235117883
UVM	B Cell	-0.166229007	0.154057766
UVM	CD8+ T Cell	0.210125911	0.06662063
UVM	CD4+ T Cell	-0.144877531	0.21178185
UVM	Macrophage	-0.052506337	0.680298173
UVM	Neutrophil	-0.209974297	0.066821757
UVM	Dendritic Cell	0.114867458	0.32979086
