# Supplementary material for: A pan‐cancer analysis of prognostic significance and immunological role of lysosomal‐associated membrane protein 3
Source: J Cell Mol Med. 2023 Dec 26;28(3):e18088. doi: 10.1111/jcmm.18088 (PMC10844704; doi:10.1111/jcmm.18088)
Supplement: Supplementary file 1 — Figure S1. [file JCMM-28-e18088-s010.pdf]

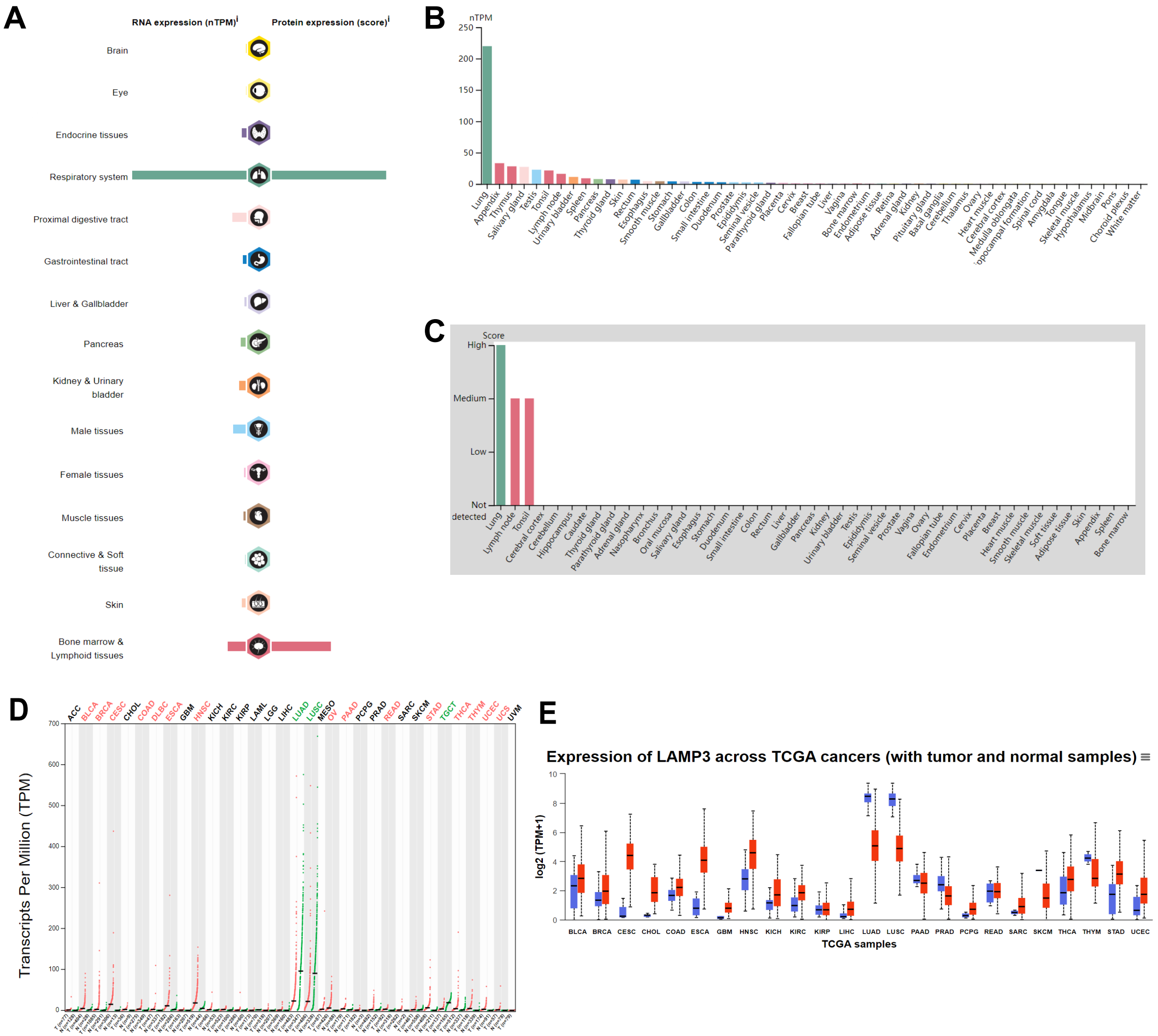
A
RNA expression (nTPM)
Protein expression (score)
Brain
Eye
Endocrine tissues
Respiratory system
Proximal digestive tract
Gastrointestinal tract
Liver & Gallbladder
Pancreas
Kidney & Urinary bladder
Male tissues
Female tissues
Muscle tissues
Connective & Soft tissue
Skin
Bone marrow & Lymphoid tissues
B
nTPM
250
200
150
100
50
0
Lung
Appendix
Thymus
Salivary gland
Testis
Tonsil
Lymph node
Urinary bladder
Spleen
Pancreas
Thyroid gland
Skin
Rectum
Esophagus
Smooth muscle
Stomach
Gallbladder
Colon
Small intestine
Duodenum
Prostate
Epididymis
Seminal vesicle
Parathyroid gland
Placenta
Cervix
Breast
Fallopian tube
Liver
Vagina
Bone marrow
Endometrium
Adipose tissue
Retina
Adrenal gland
Kidney
Pituitary gland
Basal ganglia
Cerebellum
Thalamus
Ovary
Heart muscle
Cerebral cortex
Medulla oblongata
Hippocampal formation
Spinal cord
Amygdala
Tongue
Skeletal muscle
Hypothalamus
Midbrain
Pons
Choroid plexus
White matter
C
Score
High
Medium
Low
Not detected
Lung
Lymph node
Tonsil
Cerebral cortex
Cerebellum
Hippocampus
Caudate
Thyroid gland
Parathyroid gland
Adrenal gland
Nasopharynx
Bronchus
Oral mucosa
Salivary gland
Esophagus
Stomach
Duodenum
Small intestine
Colon
Rectum
Liver
Gallbladder
Pancreas
Kidney
Urinary bladder
Testis
Epididymis
Seminal vesicle
Prostate
Vagina
Ovary
Fallopian tube
Endometrium
Cervix
Placenta
Breast
Heart muscle
Smooth muscle
Skeletal muscle
Soft tissue
Adipose tissue
Skin
Appendix
Spleen
Bone marrow
D
Transcripts Per Million (TPM)
700
600
500
400
300
200
100
0
ACC
BLCA
BRCA
CESC
CHOL
COAD
DLBC
ESCA
GBM
HNSC
KICH
KIRC
KIRP
LAML
LGG
LIHC
LUAD
LUSC
MESO
OV
PAAD
PCPG
PRAD
READ
SARC
SKCM
STAD
TGCT
THCA
THYM
UCEC
UCS
UVM
E
Expression of LAMP3 across TCGA cancers (with tumor and normal samples)
log2 (TPM+1)
10
8
6
4
2
0
BLCA
BRCA
CESC
CHOL
COAD
ESCA
GBM
HNSC
KICH
KIRC
KIRP
LIHC
LUAD
LUSC
PAAD
PRAD
PCPG
READ
SARC
SKCM
THCA
THYM
STAD
UCEC
TCGA samples
